# RNA helicases mediate structural transitions and compositional changes in pre-ribosomal complexes

**DOI:** 10.1038/s41467-018-07783-w

**Published:** 2018-12-19

**Authors:** Lukas Brüning, Philipp Hackert, Roman Martin, Jimena Davila Gallesio, Gerald Ryan R. Aquino, Henning Urlaub, Katherine E. Sloan, Markus T. Bohnsack

**Affiliations:** 1 Department of Molecular Biology, University Medical Center Göttingen, 37073 Göttingen, Germany; 2 Max Planck Institute for Biophysical Chemistry, Bioanalytical Mass Spectrometry, 37077 Göttingen, Germany; 3 Institute for Clinical Chemistry, University Medical Center Göttingen, 37073 Göttingen, Germany; 4 Göttingen Centre for Molecular Biosciences, Georg-August-University, 37073 Göttingen, Germany

## Abstract

Production of eukaryotic ribosomal subunits is a highly dynamic process; pre-ribosomes undergo numerous structural rearrangements that establish the architecture present in mature complexes and serve as key checkpoints, ensuring the fidelity of ribosome assembly. Using in vivo crosslinking, we here identify the pre-ribosomal binding sites of three RNA helicases. Our data support roles for Has1 in triggering release of the U14 snoRNP, a critical event during early 40S maturation, and in driving assembly of domain I of pre-60S complexes. Binding of Mak5 to domain II of pre-60S complexes promotes recruitment of the ribosomal protein Rpl10, which is necessary for subunit joining and ribosome function. Spb4 binds to a molecular hinge at the base of ES27 facilitating binding of the export factor Arx1, thereby promoting pre-60S export competence. Our data provide important insights into the driving forces behind key structural remodelling events during ribosomal subunit assembly.

## Abstract (web summary)

Pre-ribosomes undergo numerous structural rearrangements during their assembly. Here the authors identify the binding sites of three essential RNA helicases on pre-ribosomal particles, enabling them to provide insights into the structural and compositional changes that occur during biogenesis of the large ribosomal subunit.

## Introduction

**Fig. 1:**
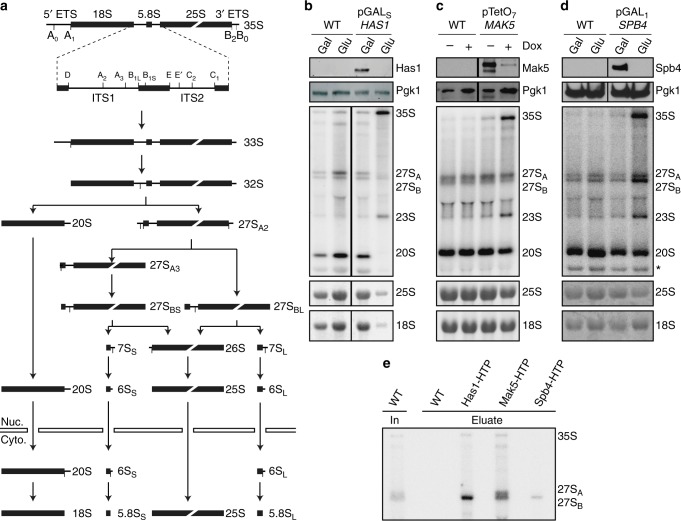
Has1, Mak5 and Spb4 are required for different steps of pre-60S maturation. **a** Schematic overview of pre-rRNA processing in *S. cerevisiae*. Mature rRNA sequences are indicated by black rectangles, and internal transcribed spacers (ITS) and external transcribed spacers (ETS) are shown as black lines. Pre-rRNA cleavage sites are marked by grey vertical lines and are named on the 35S pre-rRNA transcript. **b**–**d** Wild-type (WT) yeast, the pGAL_S_-HA-*HAS1* (**b**) and pGAL_1_-HA-*SPB4* (**d**) strain were grown in YPD (Glu) and YPG (Gal) for 12 h, and WT yeast and the pTetO_7_-HA-*MAK5* (**c**) strain were grown in the presence (+) or absence (−) of doxycycline for 10 h before harvesting. Proteins and RNAs were then extracted. Proteins were separated by SDS-PAGE and analysed by western blotting using an anti-HA antibody to detect the helicases and an anti-Pgk1 antibody as a loading control. RNAs were separated by denaturing agarose gel electrophoresis and pre-rRNAs were detected by northern blotting using probes hybridising in ITS1 and ITS2. Mature 25S and 18S rRNAs were visualised by methylene blue staining. Asterisk indicates cross-reactivity of the probes with the 18S rRNA. **e** Wild-type yeast (WT) and yeast cells expressing Has1-His_10_-TEV protease cleavage site-ProteinA (HTP), Mak5-HTP or Spb4-HTP were lysed, and complexes were retrieved on IgG sepharose and eluated by TEV protease cleavage. RNA was extracted from inputs (In; 1%) and eluates, and pre-rRNAs were detected as in (**b**–**d**)

Ribosomes are essential ribonucleoprotein complexes (RNPs) responsible for the production of all cellular proteins. The biogenesis of eukaryotic ribosomes is a complex process involving the synthesis and maturation of four ribosomal (r)RNAs, and the hierarchical recruitment and assembly of 79 ribosomal proteins in *Saccharomyces cerevisiae*^1^. Ribosome assembly is initiated in the nucleolus by RNA polymerase I-mediated synthesis of a precursor ribosomal RNA (pre-rRNA) containing the sequences of three of the four mature rRNAs flanked by external and internal transcribed spacers (ETS and ITS, respectively). A subset of ribosomal proteins and biogenesis factors are recruited to the nascent pre-rRNA transcript leading to formation of an early 90S pre-ribosomal complex. A central pre-rRNA cleavage event then separates the precursors of the small (SSU; 40S) and large (LSU; 60S) ribosomal subunits, which undergo independent maturation pathways in the nucleolus and nucleoplasm before nuclear export and final maturation steps in the cytoplasm^2^. Concurrent with protein assembly, the pre-rRNAs undergo a series of endo- and exo-nucleolytic processing events to remove the transcribed spacer regions and generate the mature rRNAs (Fig. 1a)^3^. During pre-rRNA maturation, the rRNA sequences are extensively modified by multiple small nucleolar RNPs (snoRNPs), which contain a snoRNA that basepairs with the pre-rRNA to direct 2′-*O*-methylation, pseudouridylation or acetylation of specific nucleotides, as well as by a number of stand-alone modification enzymes^4,5^.

Recent insights into the timing of ribosomal protein recruitment^6^, pre-rRNA rearrangements^7^ and structural analyses of several pre-ribosomal complexes^8–14^ reveal ribosome assembly to be highly ordered, with individual structural domains of each subunit formed sequentially. During their biogenesis, pre-ribosomal subunits undergo various structural transitions to achieve the final architecture found in mature ribosomes. For example, key events during pre-60S maturation are the docking of the 5S RNP and its 180° rotation to form the central protuberance^11^, assembly of the peptidyl transferase centre and polypeptide exit tunnel, and the removal of ITS2 sequences and biogenesis factors from the “foot” region^15,16^. Such irreversible remodelling steps, which are typically driven by NTP-dependent enzymes including GTPases, AAA-ATPases and RNA helicases, enforce the directionality of the pathway and act as key surveillance checkpoints, ensuring the fidelity of subunit assembly^17–20^.

So far, 21 RNA helicases are known to associate with pre-ribosomal complexes in yeast^20^ and functions have been ascribed to some of these proteins; several RNA helicases are implicated in the release of specific snoRNPs from pre-ribosomal complexes (Prp43, Has1, Rok1, Dhr1)^21–26^, while others are suggested to remodel pre-ribosomes to facilitate access of a pre-rRNA endonuclease (Prp43 and Nob1)^22,27^, enable rRNA modification (Kre33)^28^, drive stable incorporation of ribosomal proteins (Has1)^29^ or promote release of *trans*-acting biogenesis factors (Rok1)^30^. Knowledge of the functions and the molecular targets of other helicases remains limited but elucidation of the pre-rRNA sequences bound by the remaining pre-ribosome-associated RNA helicases and determination of their functions is key for uncovering how structural transitions during ribosome assembly are regulated.

Here we identify the pre-ribosomal-binding sites of three essential pre-60S helicases, Has1, Mak5 and Spb4 using a crosslinking-based analysis (CRAC). Our data imply a role for Has1 in unwinding U14 snoRNA-pre-rRNA duplexes and we identify a Has1 binding site on pre-60S complexes that is in line with its proposed role in driving stable incorporation of ribosomal proteins into domain I and its recruitment by a subset of factors such as Erb1^29^. We also discover crosslinking of Mak5 to 25S-H39 and structure probing data indicate that binding of the helicase to this region of the pre-rRNA promotes recruitment of the ribosomal protein Rpl10 (uL16). Finally, we identify two distinct pre-rRNA contact sites for the late-acting helicase Spb4, which may represent a binding platform and a remodelling target. Spb4 binds to the hinge region at the base of a highly flexible arm formed by H63/ES27, which anchors the pre-60S export factor Arx1^8^ and we demonstrate that Spb4 facilitates the association of Arx1 to pre-60S complexes.

## Results

### RNA helicases are required at different stages of pre-60S biogenesis

The putative RNA helicases Has1, Mak5 and Spb4 are implicated in biogenesis of the LSU^29,31,32^ and Has1 is also required for maturation of the SSU^33^. To define the pre-ribosomal complexes that Has1, Mak5 and Spb4 associate with and to demonstrate at which stage of ribosome biogenesis these proteins are required, we first established yeast strains in which each of these essential helicases could be transiently depleted by addition of doxycycline (pTetO_7_-HA-*MAK5*) or by growth in the presence of glucose (pGAL_S_-HA-*HAS1*, pGAL_1_-HA-*SPB4*) and pre-rRNAs were analysed by northern blotting to identify processing defects (Fig. 1b–d). In parallel, we isolated Has1-, Mak5- and Spb4-containing particles and investigated their pre-rRNA and protein composition using northern blotting and mass spectrometry respectively (Fig. 1e and Supplementary Table 1). These analyses revealed that Has1 associates with the initial 35S pre-rRNA transcript and 27S_B_ pre-rRNA as well as numerous ribosome biogenesis factors implicated in early SSU and intermediate LSU biogenesis (Fig. 1e and Supplementary Table 1). Lack of Has1 caused accumulation of the initial 35S pre-rRNA transcript and the aberrant 23S pre-rRNA intermediate that is often observed during cellular stress^34^, and concomitant decreases in the 20S and the 27S_A/B_ pre-rRNAs (Fig. 1b). These findings are consistent with roles for Has1 in the early steps of SSU maturation and the action of this helicase on 27S_B_ pre-rRNA-containing pre-60S complexes during LSU biogenesis. The 35S, 27S_A_ and 27S_B_ pre-rRNAs were enriched in pre-60S complexes isolated via Mak5 (Fig. 1e), suggesting that Mak5 binds upstream of Has1 during pre-60S biogenesis. This is in line with an earlier report showing that Mak5 accumulates on pre-66S complexes upon depletion of Has1^29^. Interestingly, depletion of Mak5 lead to accumulation of the 35S and 23S pre-RNAs, and decreased levels of the 27S_B_ but not 27S_A_ pre-rRNA (Fig. 1c), implying that Mak5 is recruited to early pre-60S particles, but that its presence is required when 27S_A_ is converted to 27S_B_. In line with the action of Mak5 in 27S pre-rRNA-containing complexes, a plethora of pre-60S biogenesis factors previously shown to act at this stage of LSU biogenesis, were co-precipitated with Mak5 (Supplementary Table 1). The 27S_B_ pre-rRNA was also present in Spb4-containing particles (Fig. 1e) and depletion of Spb4 was accompanied by a significant accumulation of this intermediate (Fig. 1d). This implies that is Spb4 is required for pre-60S biogenesis downstream of the pre-rRNA processing steps to remove ITS1 and that it is predominantly present in later pre-ribosomal particles than Mak5. Consistent with this, the LSU biogenesis factors identified in Spb4-containing particles are typically implicated in later steps in LSU biogenesis than those present in the Mak5 particle (Supplementary Table 1). Although the 35S pre-rRNA accumulated upon depletion of Spb4, this pre-rRNA species was not co-purified together with Spb4, suggesting that its accumulation is the result of a feedback mechanism, and in line with this model, the level of the aberrant 23S pre-rRNA also increased upon depletion of Spb4. Together, these results support a role for Has1 during early SSU biogenesis, and indicate the sequential action of Mak5, Has1 and then Spb4 during pre-60S maturation.

### The ATPase activities of Mak5 and Spb4 are required for LSU maturation

**Fig. 2:**
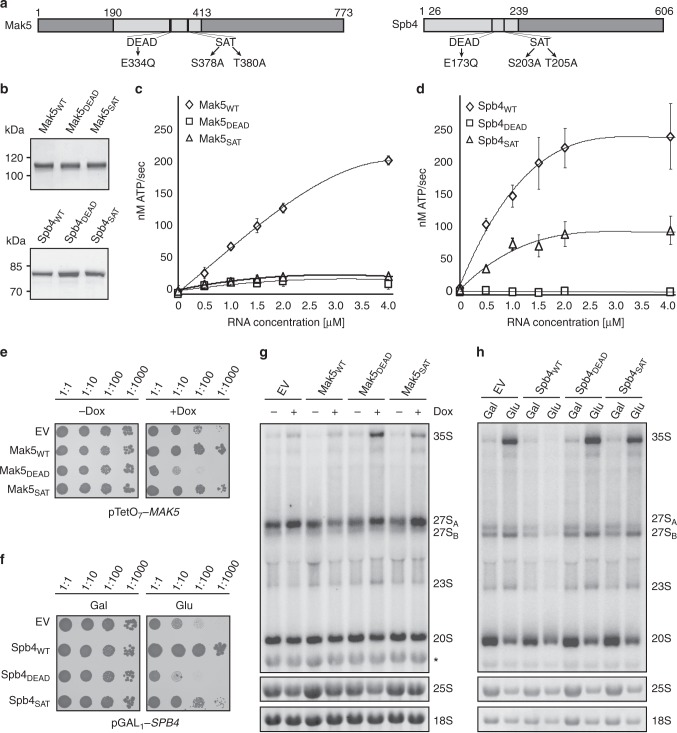
The ATPase activity of Mak5 and Spb4 is required for LSU biogenesis. **a** Schematic view of the domains of Mak5 and Spb4. The relative positions of the conserved RecA-like domains, and DEAD and SAT motifs, are highlighted. Amino acid numbers of domain boundaries are indicated above and amino acids substitutions introduced are marked below. **b** His_10_-ZZ-tagged wild-type Mak5 (Mak5_WT_), Mak5_DEAD_, Mak5_SAT_, wild-type Spb4 (Spb4_WT_), Spb4_DEAD_, Spb4_SAT_ were purified from *E. coli*, separated by SDS-PAGE and visualised by Coomassie staining. **c**, **d** The ATPase activity of recombinant Mak5 (**c**), Spb4 (**d**) and their mutants in the presence of different concentrations of RNA was monitored using NADH-coupled ATPase assays. Data from three independent experiments is shown as mean ± standard deviation. **e** The pTetO_7_*-*HA-*MAK5*–derived yeast strains carrying an empty pRS415 plasmid (EV) or pRS415-based plasmids for expression of Mak5_WT_, Mak5_DEAD_ or Mak5_SAT_ were grown in YPD before 10-fold serial dilution and growth on either YPD (-dox) or YPD supplemented with doxycycline (+dox) plates. Growth was recorded after 72 h growth at 30 °C. **f** Growth of the pGAL_1_-HA-*SPB4* yeast strain transformed with pRS415 (EV) or plasmids for expression of Spb4_WT_, Spb4_DEAD_ or Spb4_SAT_ was analysed on YPG (Gal) or YPD (Glu) as in (**e**). **g**, **h** The pTetO_7_-HA-*MAK5* and pGAL_1_-HA-*SPB4* complementation strains were grown as in (**e**, **f**) before harvesting. RNAs were isolated, separated by agarose-glyoxal gel electrophoresis and pre-rRNAs were detected by northern blotting using probes hybridising in ITS1 and ITS2. Mature rRNAs were detected by methylene blue staining. The experiments presented in (**e**–**h**) were all performed in triplicate and representative data is shown

It has previously been shown that Has1 hydrolyses ATP in an RNA-dependent manner and that its ATPase activity is required during ribosome biogenesis^29,35^. However, although both Mak5 and Spb4 possess helicase core domains (Fig. 2a), their action as ATPases has not been demonstrated so far and it remains unknown whether their catalytic activities are required for LSU biogenesis. Recombinant, His_10_-ZZ-tagged wild-type Mak5 and Spb4, or mutant versions of the helicases carrying amino acid substitutions in their conserved DEAD and SAT motifs, which are implicated in ATP hydrolysis and the coupling of ATP hydrolysis with unwinding activity respectively^36^, were purified from *E. coli* (Fig. 2a, b) and used for NADH-coupled ATPase assays. Both wild-type Mak5 and Spb4 efficiently hydrolysed ATP and the rates of ATP turnover increased at higher RNA concentrations, demonstrating that these proteins are indeed RNA-dependent ATPases (Fig. 2c, d and Supplementary Table 2). However, mutation of the DEAD motif in either protein or the SAT motif of Mak5 abolished ATPase activity and alteration of the Spb4 SAT motif diminished the ability of the protein to hydrolyse ATP (Fig. 2c, d). To investigate whether the low activity of the mutant helicases arises due to decreased interaction with the RNA substrate or impaired ATP binding/hydrolysis, the affinity of the wild-type helicases and their mutants for RNA was determined using fluorescence anisotropy (Supplementary Fig. 1 and Supplementary Table 2). Although wild-type Mak5 displayed lower affinity for RNA than wild-type Spb4, mutation of the DEAD or SAT motifs of either protein did not significantly affect their interactions with RNA, implying that their reduced catalytic activity reflects a lack of ATP binding/hydrolysis.

Having established that Spb4 and Mak5 are RNA-dependent ATPases, we next determined if their catalytic activity is required for their functions in ribosome biogenesis. This is particularly relevant as our data together with previously published work suggests that both helicases may bind to pre-60S complexes prior to the time at which they function. Yeast complementation systems were therefore generated in which pTetO_7_-HA-*MAK5* or pGAL_1_-HA-*SPB4* strains were transformed with low copy, centromeric plasmids for the expression of wild-type or mutant Mak5 or Spb4 from their endogenous promoters or the empty vector. Serial dilution assays showed that all the strains grew equally well in permissive conditions and that growth defects caused by depletion of the endogenous helicases could be rescued by expression of plasmid-derived wild-type Mak5 or Spb4 (Fig. 2e, f). Expression of either Mak5_DEAD_ or Spb4_DEAD_ lead to more significant defects than those observed when the endogenous proteins were lacking (Fig. 2e, f), demonstrating that the ATPase activities of Spb4 and Mak5 are required for their cellular functions and that disruption of their DEAD motifs can have dominant negative effects. Expression of either Mak5_SAT_ or Spb4_SAT_ lead to milder growth defects (Fig. 2e, f) and for Spb4 this is consistent with the fact that this mutant retains some catalytic activity. While mutation of the SAT motif of Mak5 significantly decreased its ATPase activity in vitro, the effect on cellular growth was not as dramatic, suggesting that this mutation may have subtly different effects in vitro and in vivo.

To determine whether the requirement for the catalytic activities of Mak5 and Spb4 for cellular growth reflects their role in ribosome biogenesis, pre-rRNA processing was analysed in the strains expressing wild-type or catalytically inactive Mak5 or Spb4. Consistent with our earlier findings, northern blot analyses of RNAs from cells depleted of Mak5 or Spb4 complemented with an empty vector showed accumulation of the 35S and the 27S_A_ (Mak5) or 27S_B_ (Spb4) pre-rRNAs compared to the non-depleted sample and these defects were rescued by re-expression of the wild-type helicases (Fig. 2g, h). Similar to depletion of the endogenous helicases, expression of Mak5 or Spb4 carrying mutations in the DEAD or SAT motifs lead to accumulations of the 35S and 27S_A_/27S_B_ pre-rRNAs (Fig. 2g, h), demonstrating that the activity, rather than the presence, of these proteins is necessary for pre-rRNA processing. Taken together, these data show that Mak5 and Spb4 are RNA-dependent ATPases and that their catalytic activity is required for their essential functions during LSU biogenesis.

### Has1 and Mak5 and Spb4 bind distinct pre-ribosomal regions

**Fig. 3:**
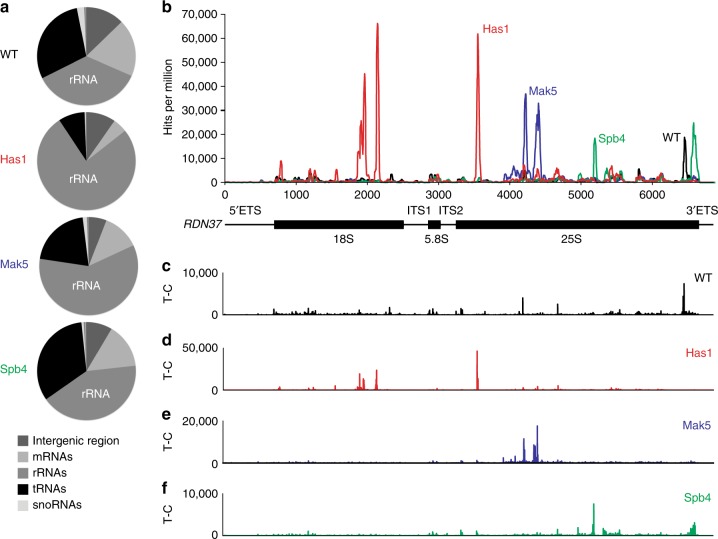
Has1, Mak5 and Spb4 crosslink to spatially distinct pre-rRNA sequences. **a** Wild-type yeast (WT) or yeast expressing C-terminally HTP tagged Has1, Spb4 or Mak5 were subjected to PAR-CRAC and the obtained sequence reads were mapped to the *S. cerevisiae* genome. The relative proportions of reads mapping to different classes of RNAs is shown. **b** For WT, Has1-HTP, Mak5-HTP and Spb4-HTP, the normalised number of reads mapped per nucleotide to the *RDN37* locus is shown above a schematic view of the initial 35S pre-rRNA transcript. Nucleotide positions on the 35S pre-rRNA transcript are indicated below the graph. **c**–**f** The number of T-C mutations detected per nucleotide of the *RDN37* locus is shown for the WT (**c**), Has1-HTP (**d**), Mak5-HTP (**e**) and Spb4-HTP (**f**) CRAC samples. ETS external transcribed spacer, ITS internal transcribed spacer

To determine the pre-rRNA targets of Has1, Mak5 and Spb4, we applied the in vivo photoactivatable ribonucleoside-enhanced crosslinking and analysis of cDNA (PAR-CRAC) approach^37,38^. Yeast strains expressing C-terminally His-TEV protease site-Protein A (HTP)-tagged Has1, Mak5 or Spb4 from their endogenous promoters or wild-type yeast were subjected to in vivo crosslinking and helicase-RNA complexes were purified under native and denaturing conditions. After partial RNase digestion, adaptors were ligated and RNAs were isolated, reverse transcribed and PCR amplified to generate cDNA libraries that were analysed by Illumina deep sequencing. The obtained sequence reads were mapped to the *S. cerevisiae* genome and reads not containing a specific T to C mutation were discarded^39^. CRAC experiments were performed at least in duplicate with high reproducibility and a representative dataset is presented. The distributions of the obtained sequence reads mapping to different classes of RNAs were then analysed (Fig. 3a and Supplementary Table 3). The proportions of reads mapping to different RNA types in the control sample reflect the distribution of non-specifically co-purified (i.e. background) RNAs that are carried through the CRAC procedure. Our analysis revealed that compared to the control, the amount of rRNA sequences retrieved with Has1, Mak5 and Spb4 were markedly increased (WT - 36%, Has1 - 76%, Mak5 - 59%, Spb4 - 42%), consistent with their involvement in ribosome assembly. The proportion of sequence reads in the Has1, Mak5 and Spb4 datasets mapping to intergenic regions, or mRNA or tRNA genes, was reduced compared to the control sample, suggesting that these helicases do not act in other aspects of RNA metabolism. Analysis of the distribution of reads mapping to the rDNA sequence encoding the 35S pre-rRNA transcript revealed specific crosslinking sites in the 25S rRNA sequence for each of the helicases as well as crosslinking sites of Has1 in the 18S rRNA sequence (Fig. 3b). The binding of a protein to these pre-rRNA sequences is further supported by the detection of specific mutations, which are introduced at nucleotides crosslinked to amino acids, in the sequence reads (Fig. 3c-f).

### Has1 crosslinks to specific sites within the 18S and 25S rRNA sequences

**Fig. 4:**
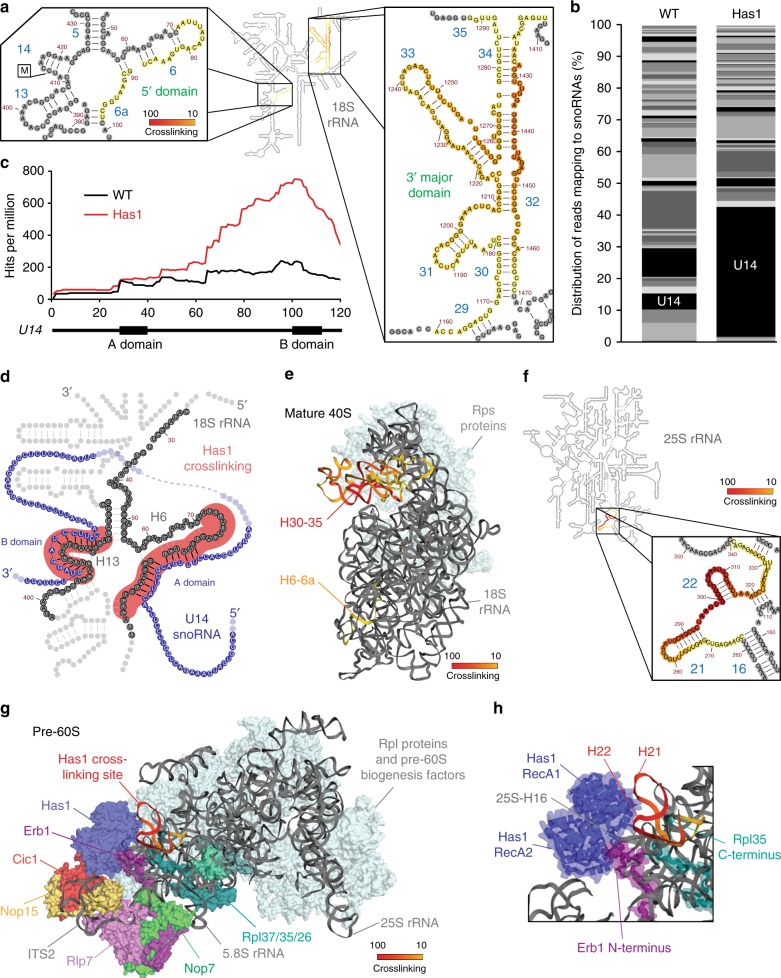
The Has1-binding sites correlate with its functions in snoRNA release and 25S-domain I assembly. **a** The number of sequence reads in the Has1-HTP CRAC data mapping to each nucleotide of the 18S rRNA sequence is shown on the secondary structure of the mature 18S rRNA using a colour code where the maximum number of reads (100%) is shown in red and lower numbers of reads (10%) are shown in yellow. Magnified views of the Has1 crosslinking sites are presented. **b** The relative number of reads mapping to each snoRNA in the WT and Has1-HTP CRAC datasets is shown. The proportion of sequence reads mapping to the U14 snoRNA is highlighted. **c** The distribution of sequence reads mapping to *U14* in the WT and Has1-HTP CRAC datasets is shown above a schematic view of the snoRNA. **d** 18S rRNA (grey) and U14 snoRNA (blue) sequences identified in chimeric reads in the Has1-HTP CRAC dataset are shown on a model of the secondary structure of the 5′ domain of the 18S rRNA. Basepairing present in the mature rRNA (grey) and between the 18S rRNA sequence and the U14 snoRNA (black) is shown. Has1 crosslinking sites are indicated in red. **e** The Has1 CRAC data was modelled onto the tertiary structure of the mature 18S rRNA (PDB: 4V88) using a colour scale as described in (**a**). **f** The Has1-HTP CRAC data was modelled onto the secondary structure of the mature 25S rRNA as in (**a**). **g** The Has1-HTP CRAC data was modelled onto the tertiary structure of the 25S rRNA in a pre-60S complex purified via Nsa1 and Nop2 (state 2; PDB: 6COF [https://www.rcsb.org/structure/6C0F]) using a colour code as in (**a**) Ribosomal proteins (Rpls) and ribosome biogenesis factors present in this particle are shown in surface view and are highlighted in difference colours as labelled. **h** A magnified view of the structural context of the Has1 crosslinking site is shown as in (**g**). Cartoon views of secondary structural features of Has1, Erb1 and Rpl35 are shown within the surface view models

Analysis of the crosslinking sites of Has1 on the secondary structure of the mature 18S rRNA^40^ revealed Has1 binding to helix (H)6/6a in the 5′ domain and to regions of H30–35 that form a stem structure in the 3′ major domain (Fig. 4a). H6/6a is a known basepairing site of the U14 snoRNA, which both forms long-range interactions within the early pre-ribosome^21,41^ and also guides 2′-*O*-methylation of 18S-C414 in H14^42^. Reads mapping across the 18S-Cm414 site were detected in the Has1 CRAC data but not the control or the CRAC data of Mak5 or Spb4, however, their relatively low number (Fig. 3b) suggests a transient interaction between Has1 and this pre-rRNA sequence. Depletion of Has1 causes accumulation of the U14 snoRNA on pre-ribosomal complexes (Supplementary Fig. 2a)^23,25^, but it has remained unknown whether the helicase is directly involved in snoRNA release. A direct role for Has1 in mediating U14 release would necessitate helicase-snoRNA interaction and we therefore examined the relative distribution of sequence reads corresponding to each of the 79 snoRNAs present in *S. cerevisiae* (Fig. 4b), which revealed a > 8-fold increase in the proportion of sequence reads mapping to the U14 snoRNA sequence in the Has1 CRAC data compared to the control, indicating a direct interaction between Has1 and U14. Analysis of the distribution of sequence reads along the U14 snoRNA revealed that the majority mapped to the U14 B domain, which basepairs with the 18S-H13-14 sequence (Fig. 4c), thereby demonstrating that Has1 contacts both the pre-rRNA and snoRNA sequences involved in U14-pre-rRNA interactions. The CRAC data were also mined for chimeric sequence reads, which can be generated if two RNA sequences are simultaneously bound by the same protein and are joined during the adaptor ligation steps, using a crosslinking and analysis of sequence hybrids (CLASH) pipeline^43^. In the Has1 CRAC data, many chimeric sequence reads composed of the U14 A domain and the 18S-H6/6a sequence were detected as well as numerous chimeras composed of the U14 B domain and the cognate 18S-H13-14 sequence containing the 18S-Cm414 modification (Fig. 4d). These data support a direct role for Has1 in release of the U14 snoRNA from its basepairing sites in the 18S rRNA sequence. Interestingly, we identified an additional Has1 crosslinking site in the 18S-H30–35 sequence, which is spatially distinct in the mature 40S structure (Fig. 4e). While this raises the possibility that Has1 has additional functions in SSU biogenesis, it is more likely that in the early pre-ribosomal complexes to which Has1 binds, the H30–35 region is in closer proximity to the 5′ domain and that U14 snoRNA release represents the primary function of Has1 during SSU biogenesis. Although Has1 was not identified in the recently published cryo-electron microscopy structures of SSU processomes^10,44,45^, the H30–35 region was reported to be highly flexible, which is consistent with this model.

To gain insight into the role of Has1 in pre-60S biogenesis, the Has1 CRAC data was mapped onto the secondary structure of the 25S rRNA, which highlighted Has1 crosslinks in H21–22 of the 25S rRNA sequence (Fig. 4f). While this manuscript was under review, cryo-electron microscopy (EM) structures of late nucleolar pre-60S particles, which contain density identified as Has1 were published^9,13,14^. Mapping of the Has1 CRAC data onto these structures (see for example PDB 6COF [https://www.rcsb.org/structure/6C0F]^13^; Fig. 4g) revealed the close proximity of the Has1 crosslinking site in H21–22 to the reported positions of Has1. Interestingly, in these structures, H21–22 lies adjacent to the RecA1 domain of Has1 suggesting that this is likely the region of the protein that is crosslinked to RNA during PAR-CRAC (Fig. 4h). The identification of a Has1 binding site in domain I of the 25S rRNA sequence is in line with a previous report showing that a subset of pre-60S biogenesis factors (Erb1, Nop7, Ytm1, Rlp7, Nop15 and Cic1), which bind to domain I, ITS2 and the 5.8S rRNA sequence, are required for Has1 recruitment into pre-60S particles^29^. Similarly, the Has1 binding site is consistent with the observation that depletion of Has1, or expression of Has1 carrying mutations predicted to impair its catalytic activity, impedes the stable assembly of the ribosomal proteins Rpl26 (uL24), Rpl35 (uL29) and Rpl37 (eL37) into domain I (Fig. 4g)^29^.

### Mak5 crosslinks to the Rpl10 binding site within the 25S rRNA sequence

**Fig. 5:**
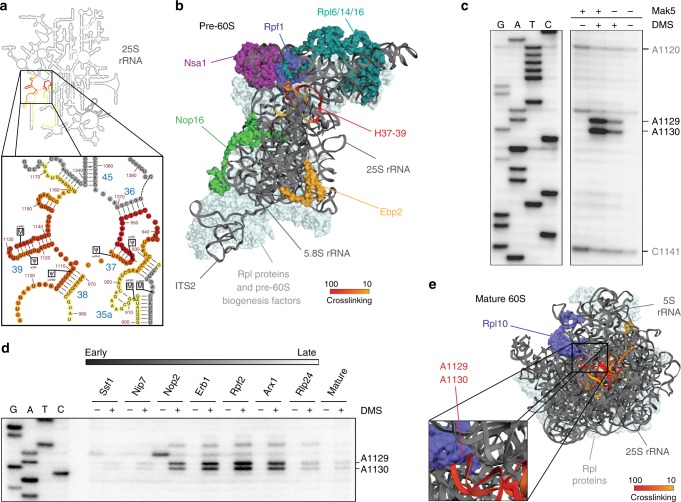
Mak5 crosslinks to the 25S rRNA sequence that is later bound by Rpl10. **a** The number of sequence reads in the Mak5-HTP CRAC data corresponding to each nucleotide of the 25S rRNA sequence is shown on the secondary structure of the mature 25S rRNA using a colour code where the maximum number of reads (100%) is shown in red and lower numbers of reads (10%) are shown in yellow. A magnified view of the Mak5 crosslinking sites in H36–37 and H39 is shown. **b** The number of sequence reads in the Mak5-HTP CRAC data corresponding to each nucleotide of the 25S rRNA sequence was modelled onto the tertiary structure of the 25S rRNA sequence in a pre-60S complex purified via Nsa1 and Nop2 (state 3; PDB: 6CB1 [https://www.rcsb.org/structure/6CB1]) using a colour code as in (**a**). Densities corresponding to specific ribosomal proteins and pre-60S biogenesis factors are highlighted in various colours. **c** Nop2-containing pre-60S complexes purified from yeast expressing (+) or depleted (−) of Mak5 were immobilised on IgG sepharose and either treated with DMS (+) or left untreated (−). RNAs isolated from these complexes served as substrate for primer extension reactions and cDNA fragments were separated by denaturing PAGE, alongside a sequencing ladder, before visualisation. Differentially methylated nucleotide are indicated. Replicate experiments were performed and representative data is presented. **d** Mature 25S rRNA and pre-60S complexes isolated via different TAP or HTP-tagged biogenesis factors that bind sequentially to early, intermediate and late pre-ribosomal complexes as indicated were immobilised on IgG sepharose and then either treated with DMS (+) or left untreated (−). RNAs isolated from these complexes were used for primer extension reactions, and cDNA fragments were separated by denaturing PAGE and visualised using a phosphorimager. **e** The number of sequence reads in the Mak5-HTP CRAC data corresponding to each nucleotide of the 25S rRNA sequence was modelled onto the tertiary structure of the mature 60S subunit (PDB: 4V88 [https://www.rcsb.org/structure/4V88]) using a colour code as in (**a**). The ribosomal protein Rpl10 is highlighted in blue and a magnified view of the contacts between 25S-H39 and Rpl10 is shown

The function(s) of Mak5 during pre-60S biogenesis have remained elusive but genetic interactions of Mak5 with a number of pre-60S biogenesis factors (Nsa1, Ebp2, Nop16, Rpf1) and ribosomal proteins (Rpl6 (eL6), Rpl14 (eL14), Rpl16 (uL13)) have been reported^46^. To visualise the structural context of the newly identified crosslinking sites, the number of sequence reads in the Mak5 CRAC data mapping to each nucleotide of the 25S rRNA sequence was modelled onto the secondary structure of the mature 25S rRNA, which revealed that the Mak5 crosslinking sites are in domain II of the 25S rRNA sequence in H36–37 and H39 (Fig. 5a). Mak5 is not present in any of the currently available cryo-EM structures of pre-60S particles but as the helicase was previously co-purified with Nsa1^46^, the Mak5 CRAC data was mapped onto the tertiary structure of a pre-60S complex isolated via the biogenesis factors Nsa1 and Nop2, which is likely to closely resemble the in vivo substrate of Mak5 (PBD 6CB1 [https://www.rcsb.org/structure/6CB1]; Fig. 5b)^13^. With the exception of the N-terminal region of Rpl6, the proteins reported to genetically interact with Mak5 are somewhat distant to the Mak5 crosslinking site (Fig. 5b), implying that the genetic interactions do not reflect physical interactions but rather that structural changes induced by Mak5 binding to domain II influence the recruitment, function or release of these factors.

Next, as the Mak5-binding sites identified contained the basepairing sites of several snoRNAs (snR5, snR8, snR43, snR51, snR60 and snR82), we analysed the levels of all 79 snoRNAs on pre-ribosomes upon depletion of Mak5 using quantitative PCR. Depletion of Mak5 did not lead to significant changes in the levels of any snoRNAs on pre-ribosomal complexes (Supplementary Fig. 2), indicating that, in contrast to Has1 (Supplementary Fig. 2)^23,25^, Mak5 does not regulate snoRNP dynamics on pre-ribosomal complexes.

To gain functional insight into the role of Mak5 and the potential changes its action may cause in the pre-rRNA structure, we monitored 25S-H36–39 by dimethyl sulfate (DMS) structure probing upon depletion of the helicase. Treatment of RNA with DMS leads to *N*^3^- and *N*^1^- methylation of accessible (non-basepaired and non-protein-bound) cytidine and adenosine residues respectively^47^, which impedes the progress of a reverse transcriptase, allowing changes in nucleotide accessibility to be monitored by primer extension. As the crosslinking sites of Mak5 lie within the sequence of the mature 25S rRNA, analysis of changes in pre-rRNA structure caused by depletion of Mak5 necessitates isolation of pre-60S complexes. Western and northern blotting were used to confirm the presence of Mak5 and the 27S_A/B_ pre-rRNAs it binds in pre-60S particles purified via Nop2 (Supplementary Fig. 3a, b). As Mak5 acts at the 27S_A_ to 27S_B_ transition (Fig. 1c), we further verified that depletion of Mak5 does not significantly affect the amount of these pre-rRNA co-purified with Nop2 (Supplementary Fig. 3c). Then Nop2-containing pre-60S complexes purified from cells expressing or depleted of Mak5 were immobilised on IgG sepharose and either treated with DMS or left untreated. Purified RNAs were used for primer extension reactions using a [^32^P]-labelled primer hybridising downstream of 25S-H39 and the obtained cDNA fragments were separated by denaturing PAGE alongside a sequencing ladder to enable identification of nucleotides where the reverse transcriptase stalled (Fig. 5c). Comparison of the modification pattern observed in DMS-treated RNAs obtained from cells expressing Mak5 revealed that in the Nop2 particle, 25S-A1129 and 25S-A1130 are readily accessible (Fig. 5c). Upon depletion of Mak5, the methylation of these residues, but not other residues such as 25S-A1120 and 25S-C1141, was significantly reduced, indicating that the presence of Mak5 contributes to the accessibility of these specific residues. This suggests that during early stages of pre-60S biogenesis, these residues may be involved in different basepairing interactions to those observed in the mature 25S rRNA structure or alternatively, that these nucleotides are bound by an early pre-60S biogenesis factor that is released by the binding of Mak5.

To test the hypothesis that the DMS-accessibility of 25S-A1129 and 25S-A1130 is altered during pre-60S biogenesis, we performed affinity purifications using HTP- or TAP-tagged pre-60S biogenesis factors as baits. While this approach leads to the isolation of heterogenous mixtures of particles, the selection of several factors that predominantly enrich different populations of pre-60S complexes, or are known to associate with exclusively early (e.g. Ssf1) or late (e.g. Arx1) pre-60S complexes enabled us to visualise the accessibility of 25S-A1129 and 25S-A1130 along the time course of pre-60S maturation. These particles were either DMS treated or left untreated and primer extension analysis was used to monitor the extent of methylation of 25S-A1129 and 25S-A1130 (Fig. 5d). Consistent with our model, in early pre-60S complexes, DMS-mediated methylation of 25S-A1129 and 25S-A1130 was low, whereas in the intermediate pre-60S complexes, methylation of these residues was significantly increased. The high accessibility of 25S-A1129 and 25S-A1130 observed in the intermediate pre-60S complexes is consistent with the lack of pre-rRNA-pre-rRNA and pre-rRNA–protein interactions formed by these nucleotides in the available pre-ribosome structures of complexes at this stage of pre-60S maturation^8,9,11–14,18^. Interestingly, in predominantly cytoplasmic pre-60S particles purified via Rlp24 and in the mature 25S rRNA, the accessibility of 25S-A1129 and 25S-A1130 was markedly reduced again. As these residues are not involved in basepairing interactions in mature ribosomes^48^, this implies that this pre-rRNA region is ultimately protected by a ribosomal protein or ribosome-associated factor. Indeed, inspection of the interactions formed by these residues in mature 60S subunits revealed that they are directly contacted by Rpl10, a ribosomal protein that is recruited late to cytoplasmic pre-60S complexes (Fig. 5e). As Mak5 and Rpl10 bind to the same pre-rRNA region, to determine whether Mak5 contributes to the recruitment of Rpl10, pre-60S complexes were enriched from cells expressing or depleted of Mak5 via the cytoplasmic biogenesis factor Kre35 and the level of Rpl10-GFP was determined by western blotting. As depletion of Mak5 affects pre-60S maturation, we verified that pre-60S particles could be exported to the cytoplasm when Mak5 is lacking and the amount of Kre35-TAP particles analysed was normalised according to the level of Rpl15 (eL15), which is incorporated during the early steps of pre-60S maturation. This revealed that upon depletion of Mak5, the amount of Rpl10 present on cytoplasmic pre-60S complexes is reduced compared to that detected in cells expressing Mak5 (Supplementary Fig. 4). Our data therefore suggest that binding of Mak5 to 25S-H37–39 of the 25S rRNA sequence during pre-60S biogenesis is required to form a structure that can be bound by Rpl10.

### Spb4 binds helix 101 and a hinge region at the base of ES27

**Fig. 6:**
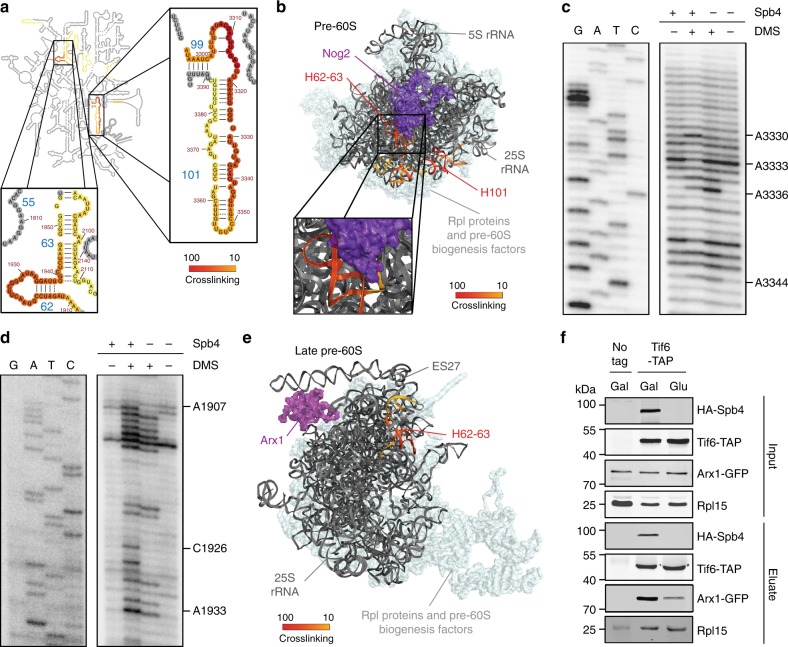
Spb4 contacts H101 and H62–63 of the 25S rRNA sequence. **a** The number of sequence reads in the Spb4-HTP CRAC data corresponding to each nucleotide of the 25S rRNA sequence is shown on the secondary structure of the mature 25S rRNA using a colour code where the maximum of reads (100%) is shown in red and lower numbers of reads (10%) are shown in yellow. Magnified views of the Spb4 crosslinking sites in H101 and H62–63 are presented. **b** The Spb4-HTP CRAC data was modelled onto the tertiary structure of the 25S rRNA sequence in a pre-60S complex purified via Nog2 (PDB: 3JCT [https://www.rcsb.org/structure/3JCT]) using a colour code as in (**a**). The position of Nog2 is indicated in purple. **c**, **d** Nop2-containing pre-60S complexes purified from yeast either expressing (+) or depleted (−) of Spb4 were immobilised on IgG sepharose and either treated with DMS (+) or left untreated (−). Isolated RNAs were used in primer extension reactions using primers hybridising downstream of H101 (**c**) or H62–63 (**d**). cDNA fragments were analysed by denaturing PAGE and visualised using a phosphorimager. Nucleotides that are differentially methylated by DMS in the presence and absence of Spb4 are indicated. Experiments were performed in replica and representative images are presented. **e** The Spb4 CRAC data was modelled onto the tertiary structure of the 25S rRNA sequence in a pre-60S complex purified via Rix1 (PDB: 5JCS [https://www.rcsb.org/structure/5JCS]) using a colour code as in (a). The position of Arx1 is indicated in purple. **f** Pre-60S complexes were isolated via TAP-tagged Tif6 from yeast expressing Arx1-GFP in the presence (Gal) or absence (Glu) of Spb4. Proteins in input and eluate samples were separated by SDS-PAGE and analysed by western blotting to detect the indicated proteins

Mapping of the Spb4 CRAC data on the secondary structure of the mature 25S rRNA revealed two crosslinking sites on the 25S rRNA sequence in H101 in domain VI and H62–63 in domain IV (Fig. 6a). While visualisation of the Spb4 CRAC data on the tertiary structure of pre-60S complexes purified via Nog2^12^ or a stalled pre-60S intermediate purified via Ytm1_E80A_^9^ confirmed that these sites are in relatively close proximity during the intermediate steps of pre-60S biogenesis, these Spb4 crosslinking sites appear to represent distinct contact sites between the helicase and pre-ribosome (Fig. 6b and Supplementary Fig. 5). Although the position of Spb4 in the Ytm1_E80A_ complex was not reported^9^, unassigned density in this particle was later demonstrated to correspond to Spb4^13^. Importantly, this structural information places Spb4 in close proximity to H101 and H62–63, confirming the crosslinking sites identified by CRAC as *bona fide* protein binding sites. Notably, no snoRNA-guided rRNA modifications are present within the crosslinking sites of Spb4 and lack of Spb4 did not significantly affect snoRNA levels on pre-ribosomal complexes (Supplementary Fig. 2), which is consistent with the requirement for Spb4 during later stages of pre-60S biogenesis, after the majority of snoRNAs have been released.

To analyse the structure of the pre-rRNA regions crosslinked by Spb4 in the presence and absence of the helicase, DMS structure probing was again employed. Based on the structural analysis described above^13^, Spb4 is anticipated to be present in pre-60S complexes containing Nop2 (Supplementary Fig. 5a). After verifying this association under our purification conditions (Supplementary Fig. 5b), Nop2-containing pre-60S complexes purified from cells expressing or lacking Spb4 were either treated with DMS or left untreated. Isolated RNAs served as templates for primer extension reactions using [^32^P]-labelled primers that anneal to the 25S rRNA sequence in H101 or H61 to examine changes in the accessibility of nucleotides within the two Spb4 crosslinking sites. Analysis of the cDNA fragments obtained using the primer annealing downstream of the Spb4 crosslinking site in H101 revealed that 25S-A3336 is more readily modified by DMS when Spb4 is lacking (Fig. 6c). Furthermore, several other nucleotides within this region of the 25S rRNA sequence showed altered DMS-modification patterns upon depletion of Spb4, whereas the extent of modification of nucleotides more distant from this Spb4 crosslinking site remained unaffected, confirming 25S-H101 as a binding site of Spb4 on the 25S rRNA. In contrast, monitoring changes in 25S-H62–63 upon depletion of Spb4 highlighted several nucleotides (A1907, C1926 and A1933), which were less accessible for DMS-mediated modification when Spb4 was lacking (Fig. 6d), implying that Spb4 is involved in structural rearrangements in this region of domain IV. Taken together with the lesser extent of crosslinking of Spb4 to 25S-H62–63 compared to 25S-H101, these data imply that domain VI may serve as a binding platform for the helicase while a transient interaction of Spb4 with 25S-H62–63 of domain IV likely reflects its remodelling activity.

### Spb4 is required for recruitment of the pre-60S export factor Arx1

Spb4 is required for assembly of the GTPase Nog2, which acts as a placeholder for the essential export adaptor Nmd3, into pre-60 complexes^17,49^. Structural analyses show that Nog2 is positioned within domain IV of pre-60S complexes (Fig. 6b), consistent with a model in which structural rearrangements around 25S-H62–63 initiated by Spb4 facilitate recruitment of Nog2 to pre-ribosomal complexes. Interestingly, the crosslinking site of Spb4 in 25S-H62–63 is at the base of eukaryotic expansion segment (ES)27, which, in late nucleoplasmic pre-60S complexes, forms a flexible arm that clamps the export factor Arx1 (Fig. 6e). The relative positions of Spb4 and Arx1 on pre-60S complexes raised the possibility that 25S-H62–63 acts as a molecular hinge and that the action of Spb4 is required for incorporation of Arx1 into pre-60 complexes. To test this hypothesis, late pre-60S complexes were purified via TAP-tagged Tif6, which is co-exported with pre-60S particles, from cells expressing or depleted of Spb4, and Arx1-GFP levels were monitored by western blotting. As lack of Spb4 affects LSU biogenesis, to enable Arx1 levels on pre-60S complexes purified from cells expressing or depleted of Spb4 to be compared, the amounts of particles were first normalised according to the Tif6 bait protein and the ribosomal protein Rpl15, which is incorporated into early pre-60S complexes (Fig. 6f). Arx1-GFP was not retrieved from control cells expressing untagged Tif6, and while Arx1-GFP was abundant in pre-60S complexes purified in the presence of Spb4, depletion of Spb4 markedly reduced the amount of Arx1, demonstrating the requirement for Spb4 for Arx1 recruitment to pre-60S complexes.

## Discussion

The production of ribosomes is an essential, but highly complex and energetically expensive, cellular process. Early proteomic analyses of yeast pre-ribosomal particles provided inventories of >200 ribosome biogenesis factors. A major challenge in the subsequent years has been to dissect the dynamic interactions of these proteins with pre-ribosomal complexes and to elucidate their precise contributions to the maturation pathway. Momentous advances in cryo-EM have recently enabled the first structures of mature ribosomes and pre-ribosomal subunits to be solved^8–14^, providing a wealth of information about the architecture of these complexes and the interactions of a subset of ribosome biogenesis factors. Already, these structural views of pre-ribosomal complexes have enabled fundamental aspects of eukaryotic ribosome assembly to be visualised, such as the modular assembly of SSU processome complexes and early pre-ribosomal particles, the sequential compaction and stabilisation of defined domains/regions, and the progressive release of biogenesis factors, concomitant with formation of structural features present in mature ribosomal subunits. They have further highlighted important structural transitions that take place during subunit assembly and understanding how these remodelling events are regulated has emerged as a key question. Due to their well-known functions in structural rearrangements of RNA and RNPs, RNA helicases likely act as important regulators of such pre-ribosomal events^20^. Here, we have identified the pre-ribosomal binding sites of three RNA helicases, Has1, Mak5 and Spb4. In the case of Has1 and Spb4, our crosslinking data are highly compatible with the positions of these proteins within recently published structures of pre-60S complexes^9,13,14^. Determining the pre-ribosomal binding sites of these enzymes has provided new insight into their recruitment, the potential targets of their remodelling activity and their functions.

Interestingly, accumulation of the U14 snoRNA on pre-40S complexes has not only been observed upon depletion of Has1^25^, but also in cells lacking another SSU helicase, Dbp4^26^, raising the question of whether these observations reflect direct or indirect effects. Our identification of Has1 crosslinking to both the U14 sequences and the 18S rRNA regions involved in basepairing interactions, together with the abundance of chimeric sequence reads derived from these sequences in the Has1 CRAC data, provides strong evidence for a direct role of Has1 in unwinding U14 snoRNA-pre-rRNA duplexes. In contrast, the U14 snoRNA does not co-precipitate with Dbp4^50^, suggesting that the observed U14 accumulation upon Dpb4 depletion is an indirect effect and that the dynamics of U14 within early pre-ribosomes can be influenced by other aspects of SSU assembly. Consistent with this model, depletion of another non-enzymatic SSU biogenesis factor Efg1 has recently been shown to perturb assembly of the 5′ domain of the 18S rRNA leading to accumulation of the U14 snoRNA on pre-ribosomes^51^. Notably, the RNA helicases Prp43 and Dhr1, which are implicated in release of a cluster of snoRNAs from pre-60S complexes and dissociation of the U3 snoRNA from its basepairing sites in the central pseudoknot region of the pre-18S rRNA sequence^22,24^, are both DEAH-box helicases that likely act by ATP-coupled, processive duplex unwinding. In contrast, as a DEAD-box helicase, Has1 performs local strand unwinding, suggesting that alternative molecular mechanisms can be employed to promote disassembly of snoRNP complexes from pre-ribosomes.

The detection of another crosslinking site of Has1 in domain I of the 25S rRNA, is in line with a proposed function in driving assembly of this pre-rRNA region by promoting the stable incorporation of several ribosomal proteins, including Rpl26, Rpl35 and Rpl37^29^. The newly available atomic resolution models of Has1 bound to pre-60S complexes give insights into its pre-rRNA contacts and provide a platform from which to consider the precise target of the remodelling action of this helicase. Mapping of the Has1 CRAC data onto these structures suggests that the crosslinking site in H21–22 reflects contacts formed by the RecA1 domain, whereas the adjacent H16 protrudes into the cleft between the two RecA-like domains of the helicase. While this suggests that 25S-H16 may be the target of Has1 activity, comparison with a crystal structure of an RNA-bound form of the mitochondrial DEAD-box protein Mms116 indicates that the RNA-protein contacts formed by Has1 and 25S-H16 are different, leading to the suggestion that this position represents a docking conformation rather than an active orientation^14^. Given the functional evidence for a role of Has1 in domain I assembly^29^ and as the available cryo-EM structures represent specific snapshots of pre-60S biogenesis, it is likely that only subtle structural rearrangements bring Has1 into its active conformation on the pre-rRNA.

The contribution of the multifunctional RNA helicase Has1 to the maturation of both ribosomal subunits as well as the observation that RNA helicases largely interact with the sugar-phosphate backbone of their substrates rendering their RNA interactions sequence independent, raises the question of how Has1 is specifically recruited to its pre-ribosomal targets. A subset of pre-60S biogenesis factors (Erb1, Nop7, Rlp7, Nop15 and Cic1) that bind in close proximity to Has1 are required for the association of the helicase and Has1 is directly contacted by Erb1 and Cic1. However, it remains unclear whether Has1 recruitment is mediated by protein–protein interactions or whether the assembly of the factors leads to formation of an RNA structure that can be bound by Has1. Interestingly, we also identified a Has1 cross-linking site in 18S-H30–35 that is distinct from its active target site in the 5′ domain of the 18S rRNA sequence; it is unlikely that this reflects two independent Has1 pre-40S binding events, suggesting that contact with 18S-H30–35 is not formed by the catalytic site of Has1 but instead that this pre-rRNA region may represent a binding platform.

CRAC analysis of Spb4 similarly revealed two crosslinking sites in 25S-H63 and 25S-H101, which we suggest may reflect a remodelling site and a binding platform for the helicase respectively. Our data implicate Spb4 in structural rearrangement of 25S-H63 that forms a hinge region at the base of ES27 to enable recruitment of the pre-60S export factor Arx1. Interestingly, Nog2, which binds in close proximity to Spb4 and is also dependent on the helicase for its recruitment^49^, functions as a placeholder for the essential export adaptor Nmd3^17,52^, implying that Spb4 may contribute to establishing the export competence of pre-60S particles. Interestingly, Spb4 has a highly basic C-terminal region extending beyond the conserved RecA-like domains of the helicase core. In other RNA helicases, such as Mss116, such “basic tails” have been shown to anchor the enzymes in proximity to their target duplexes^53^, raising the possibility that the crosslinking site of Spb4 in 25S-H101 reflects a pre-rRNA contact of this region of the protein. Although we only identified one crosslinking site for Mak5 in 25S-H36–39, this helicase similarly has a highly basic C-terminal extension, which has previously been shown to be important for Mak5 function and cell viability^46^. Our data suggest that binding of Mak5 to 25S-H39 during the early stages of pre-60S biogenesis makes this region accessible for the subsequent recruitment of Rpl10 after export of pre-60S complexes to the cytoplasm. Recruitment of Rpl10 is a critical step in LSU maturation as not only does it promote release of the pre-60S export factor Nmd3^54^ but also the presence of Rpl10 in mature 60S subunits is essential for subunit joining and this protein acts as a pivot for ribosome rotations during translation^55,56^. Taken together, identification of the binding sites of Has1, Mak5 and Spb4 and analysis of their functions highlights the diversity of the roles that RNA helicases can play during assembly of large ribonucleoprotein complexes.

## Methods

### Yeast strains and growth analysis

Yeast strains are based on the BY4741 strain^57^ and were grown in YPD (1% yeast extract, 2% peptone, 2% glucose), YPG (1% yeast extract, 2% peptone, 2% galactose) or synthetic media lacking specific amino acids (ForMedium). The endogenous promoters of *HAS1*, *MAK5* or *SPB4* were exchanged for either the pTetO_7_ promoter (*MAK5*), the pGAL_1_ promoter (*SPB4*) or the pGAL_S_ promoter (*HAS1*) using standard methods (Supplementary Tables 4 and 5)^58,59^. Insertion of the regulatable promoter was accompanied by integration of a 3xHA-tag to enable detection of the expressed protein using an anti-HA antibody. C-terminal tagging (His_10_-TEV protease cleavage site-Protein A (HTP); calmodulin-binding peptide-TEV protease cleavage site-Protein A (TAP); green fluorescent protein (GFP)) of other ribosome biogenesis factors was achieved using similar methods (Supplementary Tables 4 and 5). To deplete Mak5, the pTetO_7_-HA-*MAK5* strain was grown in exponential phase in the presence of 10 μg/ml (YPD) or 20 μg/ml (synthetic media) doxycycline for 10 h, whereas depletion of Has1 or Spb4 was achieved by growth of the pGAL_S_-HA-*HAS1* or pGAL_1_-HA-*SPB4* in YPD for 12 h. To generate complementation systems, the *SPB4* and *MAK5* coding sequences with 500 bp upstream and 500 bp downstream of the start and stop codons (to include the endogenous promoters and terminators) were cloned into the pRS415 vector to enable expression of the proteins at their endogenous levels in yeast. Site-directed mutagenesis, using the primers listed in Supplementary Table 5, was performed to alter the coding sequences of *MAK5* and *SPB4* to introduce glutamate to glutamine substitutions in the conserved DEAD motifs (Spb4 - E173Q (Spb4_DEAD_); Mak5 - E334Q (Mak5_DEAD_)) and to convert the SAT motifs to AAA (Spb4 - S203A and T205A (Spb4_SAT_); Mak5 - S378A andT380A (Mak5_SAT_)) in the expressed proteins. These plasmids were used to transform the pTetO_7_-HA-*MAK5* and pGAL_1_-HA-*SPB4* strains (Supplementary Table 4). The growth of yeast strains was monitored using serial dilution assays in which an equal number of cells from exponentially growing cultures were serially diluted then spotted onto appropriate media and colony number/size was determined after growth at 30 °C for 72 h.

### Analysis of protein levels in yeast

For analysis of protein levels in yeast, cells were pelleted, resuspended in water and lysed by vortexing in the presence of glass beads before precipitation of proteins using 15% trichloroacetic acid (TCA). Protein samples were separated by denaturing (sodium dodecyl sulfate; SDS) polyacrylamide gel electrophoresis (PAGE) and analysed by western blotting using antibodies listed in Supplementary Table 6.

### RNA extraction and northern blotting

Total RNA was isolated from exponentially growing yeast cells using phenol:chloroform extraction^60^. To detect pre-rRNAs, total RNA was denatured using glyoxal at 55 °C, separated on a 1.2% agarose gel in BPTE (10 mM PIPES, 30 mM Bis-Tris, 1 mM EDTA pH 8.0) and then transferred to a nylon membrane. Northern blotting using 5′-[^32^P]-labelled DNA oligonucleotides (Supplementary Table 5) that hybridise to ITS1 and ITS2 respectively, was performed and pre-rRNAs were visualised using a phosphorimager.

### Purification of recombinant proteins

The coding sequences of *SPB4* and *MAK5* were cloned (see Supplementary Table 5 for primer sequences) into a pQE80-derivative vector for expression of proteins with an N-terminal His_10_-ZZ tag in *Escherichia coli (E. coli)*. Site-directed mutagenesis was used, as described above, to generate constructs for expression of Spb4_DEAD_, Spb4_SAT_, Mak5_DEAD_ and Mak5_SAT_. The plasmids for recombinant expression of His_10_-ZZ-tagged wild-type or mutant Spb4 or Mak5 were used to transform BL21 Codon Plus cells and protein expression was induced by addition of 1 mM IPTG at 18 °C overnight. The cells were harvested and lysed by sonication in a buffer containing 50 mM Tris-HCl pH 7.0, 500 mM NaCl, 1 mM MgCl_2_, 10 mM imidazole, 1 mM PMSF and 10% glycerol, then centrifuged at 20,000 × g for 20 min at 4 °C to pellet cell debris. To precipitate nucleic acids, polyethyleneimine (PEI) was added to the soluble extract to a final concentration of 0.05% and the sample was incubated for 15 min at 4 °C. Following centrifugation at 33,000 × g for 30 min at 4 °C, His-tagged proteins were retrieved from the supernatant on cOmplete His-Tag purification resin (Roche). After sequential washing steps with Wash buffer I (50 mM Tris-HCl pH 7.0, 500 mM NaCl, 1 mM MgCl_2_, 30 mM imidazole, 10% glycerol), Wash buffer II (50 mM Tris-HCl pH 7.0, 1 M NaCl, 1 mM MgCl_2_, 30 mM imidazole, 10% glycerol) and Wash buffer I, proteins were eluted in 50 mM Tris-HCl pH 7.0, 500 mM NaCl, 1 mM MgCl_2_, 300 mM imidazole, 10% glycerol. Fractions containing protein were pooled, glycerol was added to a final concentration of 20% and the sample was dialysed against 50 mM Tris-HCl pH 7.0, 120 mM NaCl, 2 mM MgCl_2_, 20% glycerol overnight at 4 °C. The concentration of the purified protein was determined using a Bradford assay and proteins were visualised by SDS-PAGE followed by Coomassie staining.

### In vitro ATPase assays

ATP hydrolysis by recombinant RNA helicases was monitored using in vitro NADH-coupled ATPase assays^61^. Reactions were set up containing 45 mM Tris-HCl pH 7.4, 25 mM NaCl, 2 mM MgCl_2_, 4 mM ATP, 1 mM phosphoenolpyruvate (PEP), 20 U/ml pyruvate kinase/lactic dehydrogenase, 300 μM NADH, 2 μM helicase, 0–4 μM RNA (32 nt; 5′-GUAAUGAAAGUCCAUGUAAAACAAAACAAAAC-3′) and the absorbance at 340 nm was monitored in a BioTEK Synergy plate reader every 50 sec for 30 min. The rates of ATP hydrolysis were then determined using the Michaelis-Menten equation.

### Isolation of pre-ribosomal complexes on IgG sepharose

Yeast strains expressing TAP- or HTP-tagged ribosome biogenesis factors were grown in exponential phase before harvesting. Cells were lysed by grinding in liquid nitrogen in a buffer containing 50 mM Tris-HCl pH 7.8, 150 mM NaCl, 1.5 mM MgCl_2_, 0.1% NP-40, 5 mM β-mercaptoethanol and protease inhibitors. After centrifugation to pellet cell debris, Protein A-tagged proteins were retrieved on IgG sepharose. Following washing steps, complexes were eluted with TEV protease overnight at 4 °C. RNAs in the eluate were extracted and analysed by northern blotting as described above. Alternatively, proteins in the eluate were precipitated by addition of TCA to a final concentration of 20% and then analysed by western blotting using antibodies listed in Supplementary Table 6.

### Chemical structure probing

Pre-ribosomal complexes containing a TAP- or HTP-tagged ribosome biogenesis factor were immobilised on IgG sepharose as described above and dimethyl sulfate (DMS) was added to half of the sample to a final concentration of 0.5% for 2 min at room temperature^62^. Reactions were quenched by addition of 250 mM β-mercaptoethanol and RNAs were extracted from the IgG sepharose-bound complexes as above. Co-precipitated, DMS-treated or untreated RNAs then served as substrates in primer extension reactions using 5′-[^32^P]-labelled DNA oligonucleotides (Supplementary Table 5) and Superscript III reverse transcriptase. cDNA products were separated by denaturing (7 M urea) PAGE alongside a sequencing ladder generated by reverse transcription of total RNA in reactions containing trace amount of dideoxynucleotides. Radiolabelled cDNA fragments were detected using a phosphorimager.

### Crosslinking and analysis of cDNA (CRAC)

PAR-CRAC^21,37,63^ was performed using yeast strains expressing HTP-tagged Has1, Mak5 or Spb4 from their genomic loci. Cells were grown from an OD_600_ of 0.1 to 0.5 in low uracil media (10 mg/L uracil) supplemented with 100 μM 4-thiouracil before addition of 4-thiouracil to a final concentration of 1 mM and growth for a further 4 h^38^. Cells were then harvested and 4-thiouridine-containing RNAs were crosslinked to associated proteins at 365 nm using two cycles of 600 mJ/cm^2^. RNA-protein complexes were isolated on IgG sepharose and NiNTA and a partial RNase digest was performed using RNace-IT. After ligation of sequencing adaptors, cDNA libraries prepared by reverse transcription and PCR amplification were subjected to Illumina deep sequencing. The obtained sequence reads were mapped to the *S. cerevisiae* genome^39^ and reads not containing a T-C mutation were discarded. Python scripts were used for mapping of the obtained CRAC data onto the secondary structures of the rRNAs^40^ and tertiary structures of a mature *S. cerevisiae* ribosome (PDB: 4V88 [https://www.rcsb.org/structure/4V88])^48^, and various pre-60S complexes (PDB: 3JCT [https://www.rcsb.org/structure/3JCT]^12^, 5JCS [https://www.rcsb.org/structure/5JCS]^18^, 6ELZ [https://www.rcsb.org/structure/6ELZ]^9^, 6C0F [https://www.rcsb.org/structure/6C0F] and 6CB1 [https://www.rcsb.org/structure/6CB1]^13^). Identification of snoRNA-rRNA hybrids in the CRAC data was achieved using a bioinformatics pipeline developed for crosslinking, ligation and analysis of sequence hybrids (CLASH)^43^.

## Supplementary information

Supplementary Information

Reporting Summary

## Data Availability

The CRAC datasets of Has1, Mak5, Spb4 and the wild-type yeast control are deposited in Gene Expression Omnibus (GEO) database [http://www.ncbi.nlm.nih.gov/geo/] under the accession code GSE109216. Other data supporting the findings of this study are available from the corresponding authors upon reasonable request.
