# Supplementary material for: RNA helicases mediate structural transitions and compositional changes in pre-ribosomal complexes
Source: Nat Commun. 2018 Dec 19;9:5383. doi: 10.1038/s41467-018-07783-w (PMC6300602; doi:10.1038/s41467-018-07783-w)
Supplement: Supplementary file 1 — Supplementary Information [file 41467_2018_7783_MOESM1_ESM.pdf]

SUPPLEMENTARY INFORMATION

RNA helicases mediate structural transitions and compositional changes in pre-ribosomal complexes

L. Brüning et al.

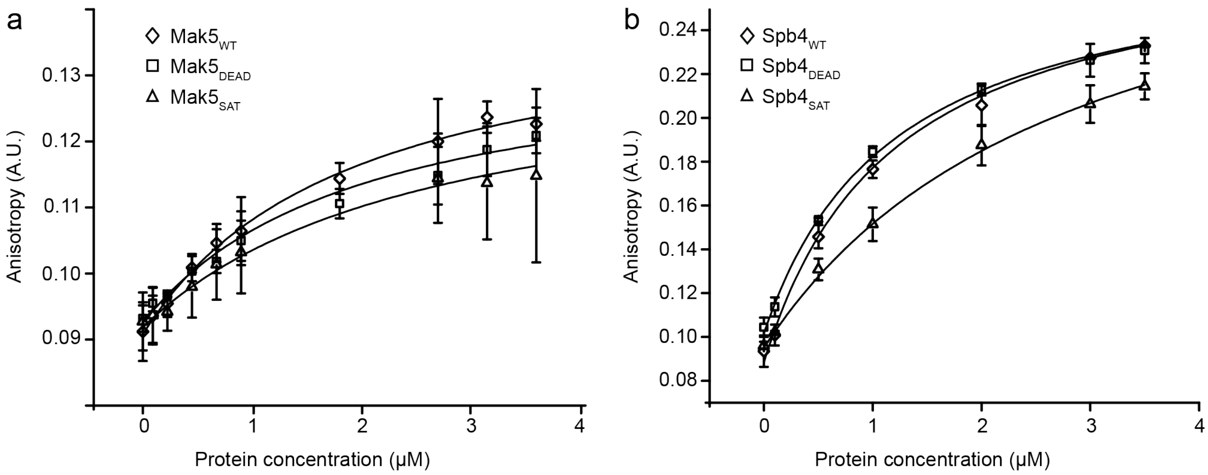

Supplementary Figure 1. RNA binding by wild-type and mutant Spb4 and Mak5. (a) Anisotropy measurements of an Atto647-labelled RNA in the presence of different amounts of wild-type Mak5 (Mak5$_{WT}$) and Mak5 carrying amino acid substitutions in the DEAD (Mak5$_{DEAD}$) or SAT motifs (Mak5$_{SAT}$). (b) Anisotropy experiments were performed as in (a) using wild-type Spb4 (Spb4$_{WT}$) and Spb4 carrying amino acid substitutions in the DEAD (Spb4$_{DEAD}$) or SAT motifs (Spb4$_{SAT}$). Data from three independent experiments are shown as mean ± standard deviation.

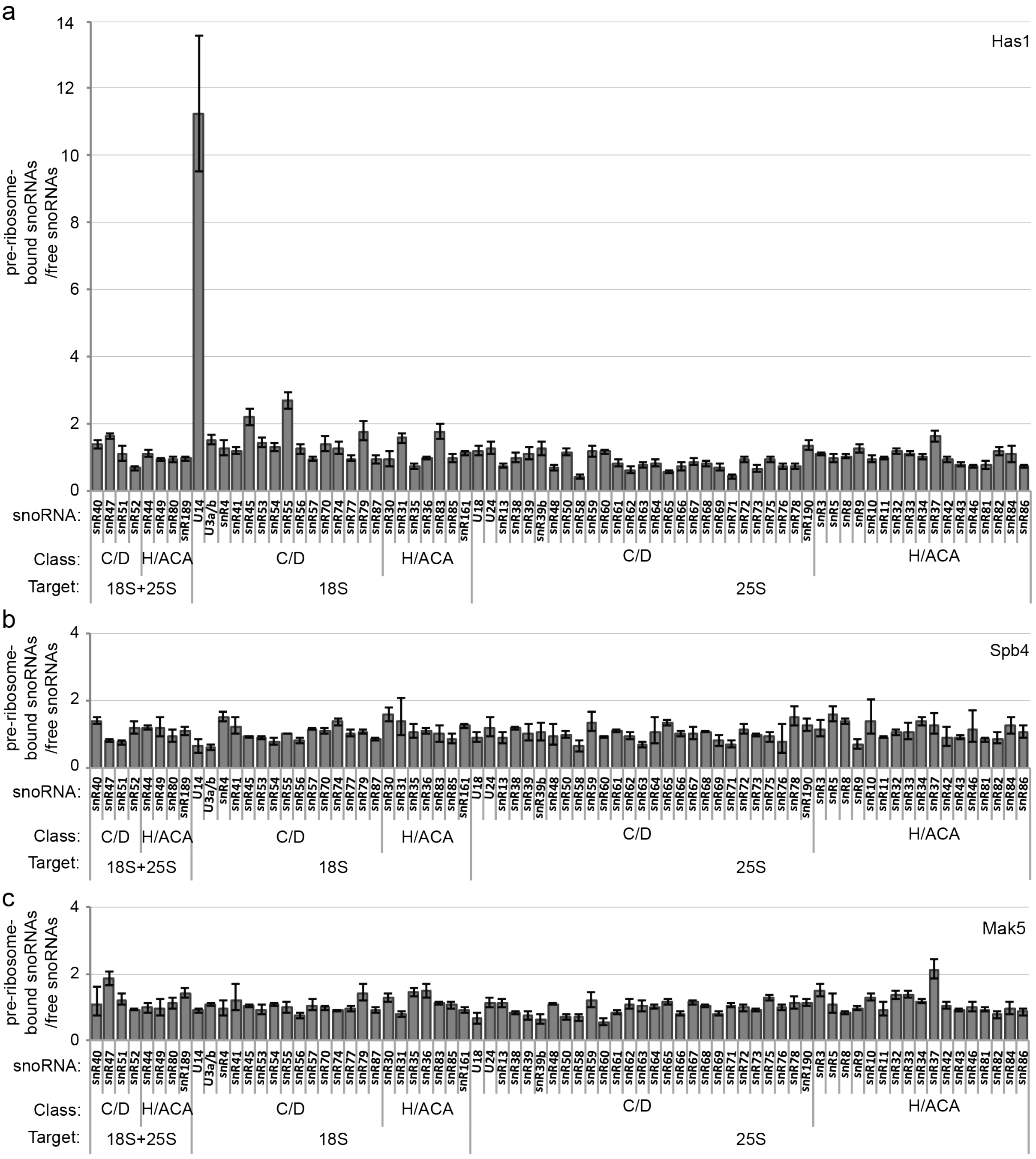

Supplementary Figure 2. Analysis of snoRNA levels on pre-ribosomes upon depletion of Has1, Spb4 or Mak5. (a-c) Wild-type yeast and the pTetO$_7$-HA-*HAS1*, pGAL$_1$-HA-*SPB4* and pTetO$_7$-HA-*MAK5* were grown in non-permissive conditions for the appropriate time to deplete each RNA helicase. Cell extracts were separated by sucrose density gradient centrifugation and fractions containing either (pre-)ribosomal complexes or non-ribosomal proteins were pooled and RNAs were extracted. After reverse transcription, the levels of 75

snoRNAs were determined by qPCR. The relative distribution of each snoRNA between the (pre-)ribosome-bound and non-ribosome-associated fractions was calculated and differences in this ratio between the wild-type yeast and upon depletion of Has1 (a), Spb4 (b) or Mak5 (c) were determined and are shown graphically. Data from three independent experiments are presented as mean ± standard deviation.

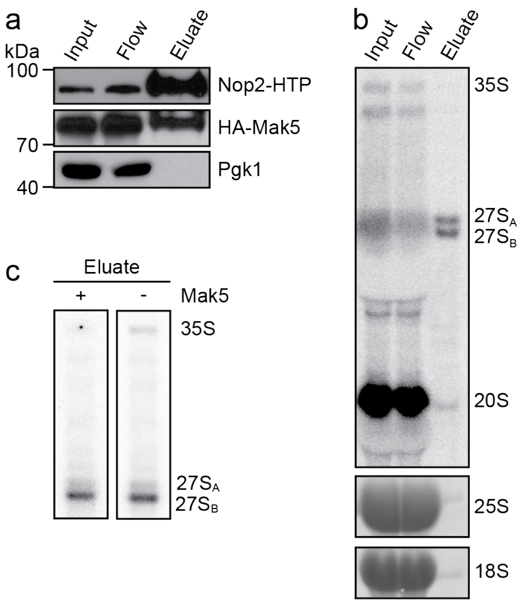

Supplementary Figure 3. Mak5 and the $27S_{A/B}$ pre-rRNAs are present in pre-60S particles purified via Nop2. (a) Yeast cells expressing HA-Mak5 and Nop2-HTP were grown in YPG before harvesting and lysis. Complexes containing Nop2-HTP were retrieved on IgG sepharose, and proteins in the input (1%), non-bound material (Flow; 1%) and the eluate were analysed by western blotting using antibodies against the Protein A tag (Nop2-HTP), the HA tag (Mak5) and, as a loading control, Pgk1. (b) Yeast cells expressing Nop2-HTP were used for a pulldown assay as in (a). RNAs isolated from the input (5%), non-bound material (Flow; 5%) and eluate were analysed by agarose-glyoxal gel electrophoresis followed by northern blotting using probes hybridising to ITS1 and ITS2 to detect pre-rRNAs. Mature 25S and 18S rRNAs were visualised by methylene blue staining. (c) The $pTetO_7$-HA-*MAK5* yeast strain expressing Nop2-HTP was grown in the presence (+) or absence (-) of doxycycline to deplete Mak5. Nop2-containing complexes were retrieved on IgG sepharose, eluted by TEV protease cleavage and pre-rRNAs in the eluate were detected as in (b).

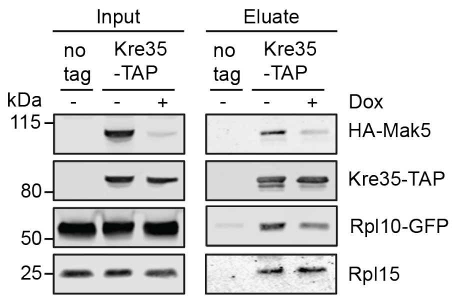

Supplementary Figure 4. Depletion of Mak5 reduces the amount of Rpl10 present on cytoplasmic pre-60S complexes. Pre-60S complexes were isolated via TAP-tagged Kre35 from yeast expressing Rpl10-GFP in the presence (- dox) or absence (+ dox) of Mak5. Proteins in input and eluate samples were separated by SDS-PAGE and analysed by western blotting to detect the indicated proteins.

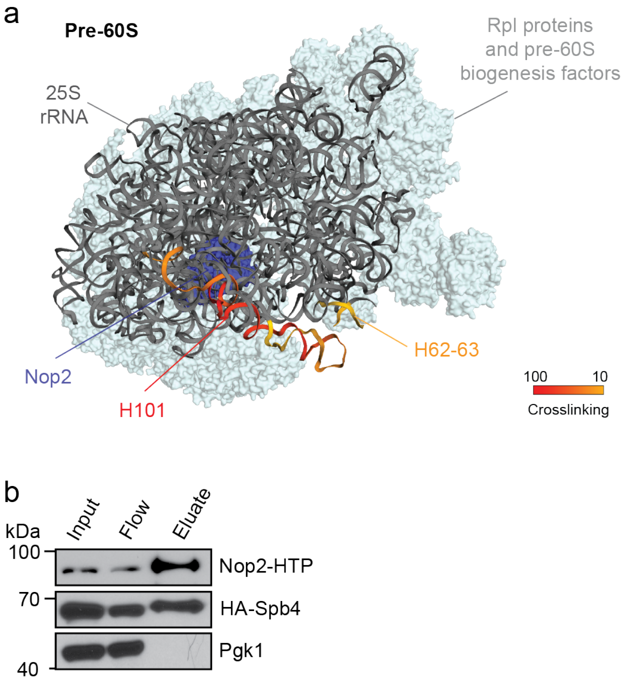

Supplementary Figure 5. Mapping of the Spb4 CRAC data on the tertiary structure of a stalled pre-60S complex purified via Rix1 and Rpf2. (a) The number of sequence reads in the Spb4-HTP CRAC data corresponding to each nucleotide of the 25S rRNA sequence was modelled onto the tertiary structure of the 25S rRNA sequence in a stalled pre-60S complex purified via $Ytm1_{E80A}$ (PDB: 6ELZ) using a colour code where the maximum number of reads (100%) is shown in red and lower numbers of reads (10%) are shown in yellow. The density corresponding to Nop2 is indicated in blue. (b) Yeast cells expressing HA-Spb4 and Nop2-HTP were grown in YPG before harvesting and lysis. Complexes containing Nop2-HTP were retrieved on IgG sepharose, and proteins in the input (1%), non-bound material (Flow; 1%) and the eluate were analysed by western blotting using antibodies against the Protein A tag (Nop2-HTP), the HA tag (Spb4) and, as a loading control, Pgk1.

Supplementary Figure 6.

Related to Figure 1b

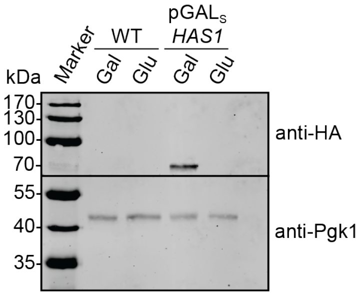

Related to Figure 1c

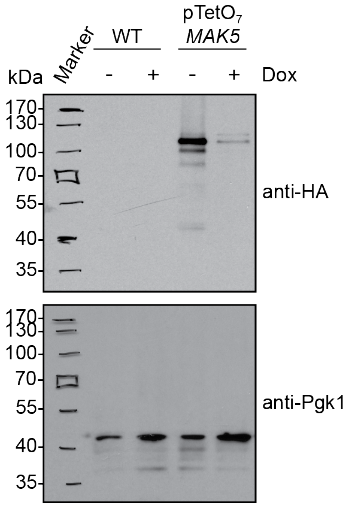

Related to Figure 1d

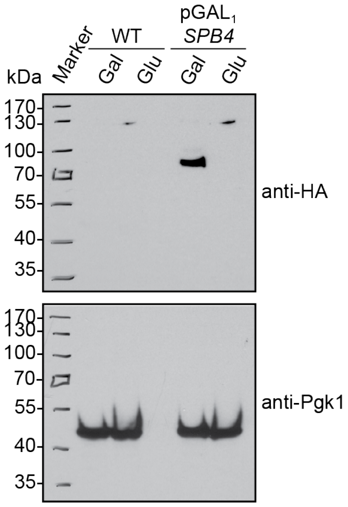

Related to Figure 2b

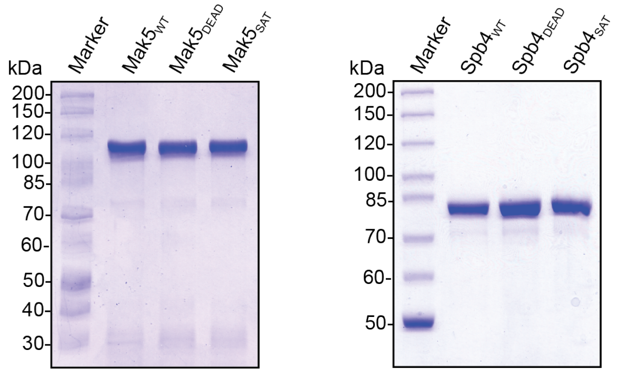

Related to Figure 6f

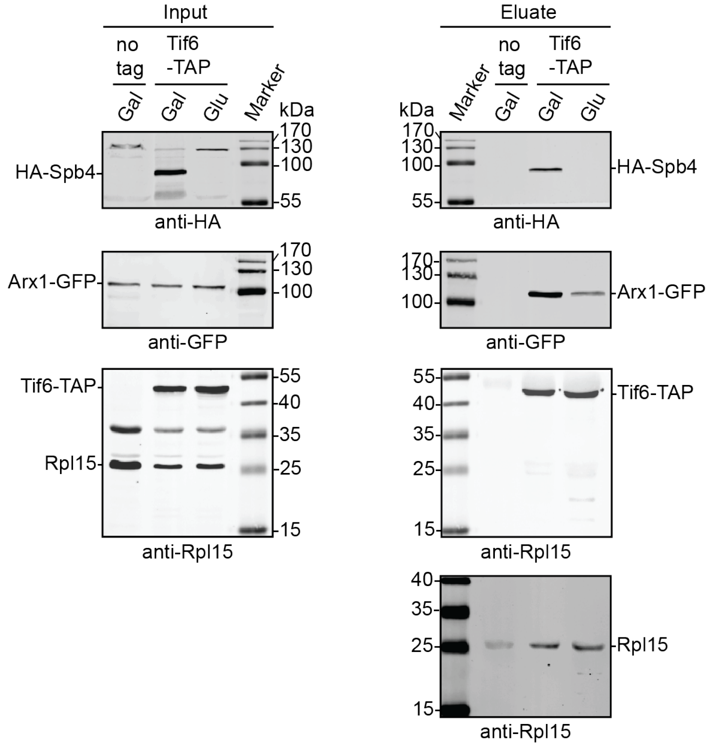

Related to Supplementary Figures 3a and 5b

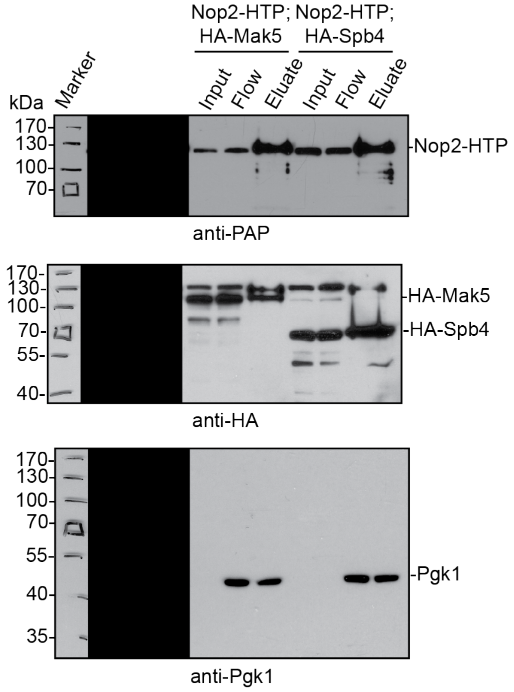

Related to Supplementary Figure 4

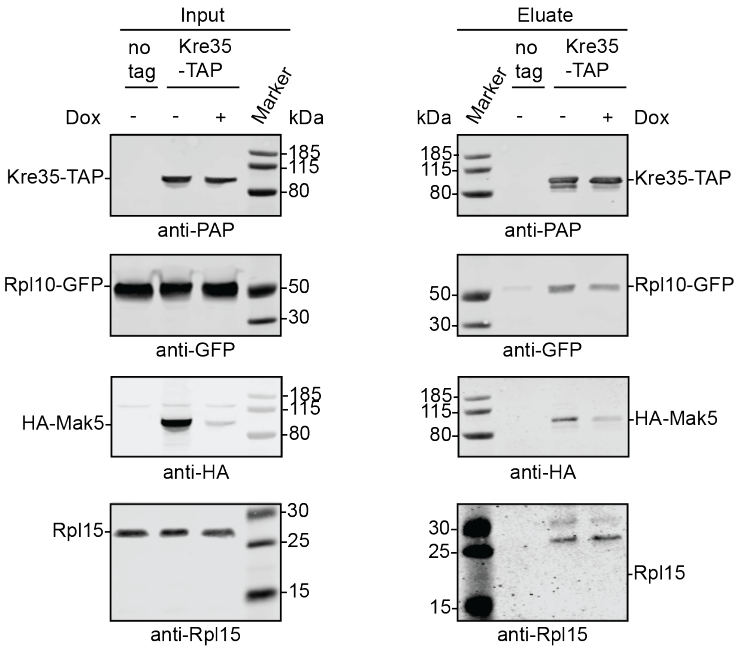

Supplementary Table 1. Ribosomal proteins and ribosome biogenesis factors co-purified with Has1, Mak5 and Spb4. Proteins > 3-fold enriched in the Has1-TAP, Mak5-TAP or Spb4-HTP eluates of two independent experiments compared to the relevant controls are listed.

Has1	Mak5	Spb4
Brx1	Bms1	Brx1
Cic1	Cic1	Cic1
Dbp10	Dbp10	Dbp10
Drs1	Dbp7	Dbp9
Ebp2	Dbp9	Ebp2
Ecm16	Ebp2	Erb1
Erb1	Erb1	Has1
Jip5	Kre33	Loc1
Loc1	Mak16	Mak5
Mak5	Nog1	Mrt4
Mrt4	Nog2	Nip7
Noc2	Nop12	Noc2
Noc3	Nop13	Noc3
Nog1	Nop14	Nog1
Nog2	Nop2	Nog2
Nop1	Nop4	Nop12
Nop13	Nop53	Nop13
Nop15	Nop7	Nop15
Nop16	Nop8	Nop16
Nop2	Nsa1	Nop2
Nop7	Pol5	Nop7
Nsa1	Puf6	Nsa1
Nsa2	Pwp1	Nsa2
Nug1	Rex4	Nug1
Puf6	Rpa135	Rpf2
Rlp7	Rpf1	Rlp7
Rpf1	Rpf2	Rlp24
Rpf2	Rpl15A	Rpl5
Rpl15a	Rpl1A	Rpl23a
Rpl1a	Rpl20A	Rpl24a
Rpl2a	Rpl21b	Rrp17
Rpl3	Rpl24a	Rsa4
Rpl4a	Rpl2A	Spb1
Rpl8a	Rpl3	Urb1
Rpl8b	Rpl4A	Utp20
Rpp0	Rpl5	Ytm1
Rps1a	Rpl8	Rpl26a
Rps4a	Rpl8a	
Rrp1	Rpp0	
Rrp15	Rps17a	
Spb1	Rps190	
Ssf1	Rps1A	
Ytm1	Rps1b	
	Rps4A	
	Rps8	
	Rrp12	
	Rrp5	
	Rrp8	

Sad1
Spb1
Spb4
Urb1
Utp10
Utp20
Utp22
Utp7
Ytm1

Supplementary Table 2. ATP hydrolysis kinetics and dissociation constants for Spb4 and Mak5, and their mutants.

Protein	K_M (μM)	k_{cat} (s^{-1})	K_d (μM)
$Mak5_{WT}$	6.00 ± 0.98	254.7 ± 28.81	2.09 ± 0.42
$Mak5_{DEAD}$	0.14 ± 0.13	9.35 ± 1.01	2.19 ± 0.37
$Mak5_{SAT}$	0.58 ± 0.27	16.20 ± 2.19	2.90 ± 0.95
$Spb4_{WT}$	0.89 ± 0.19	150.50 ± 10.96	1.16 ± 0.08
$Spb4_{DEAD}$	0.00 ± 0.19	3.69 ± 1.38	1.18 ± 0.10
$Spb4_{SAT}$	0.78 ± 0.28	59.54 ± 6.94	2.81 ± 0.43

Supplementary Table 3. Relative proportions of CRAC sequencing reads mapping to different classes of RNA.

RNA	Control (%)	Has1 (%)	Mak5 (%)	Spb4 (%)
rRNA	36	76	59	42
mRNA	19	5	12	15
tRNA	29	9	21	32
snoRNA	2	2	1	1
Intergenic regions	13	10	6	9

Supplementary Table 4. Yeast strains used in this study.

Strain	Genotype	Source
YMB 006/BY4741	MATa; his3Δ1; leu2Δ0, met15Δ0; ura3Δ0	D. Tollervey
YMB 145	YMB 279; pTetO$_7$-3xHA-*MAK5* (NatNT2)	This study
YMB 279	YMB 006; tTA::LYS2; tetR'::URA3; K.l.::LEU2	D. Tollervey
YMB 341	YMB 006; *SSF1*-TAP::HIS	Open Bios.
YMB 343	YMB 006; *ARX1*-TAP::HIS	Open Bios.
YMB 345	YMB 006; *NIP7*-TAP::HIS	Open Bios.
YMB 428	YMB 006; *HAS1*-HTP::URA3	This study
YMB 474	YMB 006; *MAK5*-HTP::URA3	This study
YMB 493	YMB 006; *SPB4*-HTP::URA3	This study
YMB 979	YMB 145; *NOP2*-HTP::HIS	This study
YMB 998	YMB 279; pGAL$_1$-3xHA-*SPB4* (KanMX); *NOP2*-HTP::HIS	This study
YMB 1097	YMB 006; pGAL$_S$-3xHA-*PRP43* (NatNT2); *RPF2*-HTP::HIS	This study
YMB 1139	YMB 006; *ERB1*-HTP::HIS	This study
YMB 1173	YMB 006; *RLP24*-HTP::HIS	This study
YMB 1453	YMB 979; pRS415::LEU2	This study
YMB 1455	YMB 979; pRS415-*MAK5*::LEU2	This study
YMB 1457	YMB 979; pRS415-*MAK5$_{DEAD}$*::LEU2	This study

YMB 1459	YMB 979; pRS415-*MAK5*$_{SAT}$::LEU2	This study
YMB 1405	YMB 998; pRS415::LEU2	This study
YMB 1407	YMB 998: pRS415-*SPB4*::LEU2	This study
YMB 1464	YMB 998; pRS415-*SPB4*$_{DEAD}$::LEU2	This study
YMB 1466	YMB 998; pRS415-*SPB4*$_{SAT}$::LEU2	This study
YMB 1613	YMB 006; pGAL$_S$-3xHA-*HAS1* (NatNT2)	
YMB 1670	YMB 006; pGAL$_1$-3xHA-*SPB4* (KanMX); *TIF6*-TAP::URA3; *ARX1*-GFP (Hyg)	This study
YMB 1702	YMB 006; *HAS1*-TAP::URA3	Eurogentec
YMB 1726	YMB 145; *KRE35*-TAP (NatNT2); pRS415-pGAL$_1$-*RPL10*-GFP::LEU2	This study
YMB 1736	YMB 006; *MAK5*-TAP (KanMX)	Eurogentec

Supplementary Table 5. Oligonucleotide primers used in this study

Primer	Sequence (5'-3')	Purpose
Spb4_fw_NcoI	ATATTACCATGGCCATGTCAAAGTCATTGGAATGGG	Cloning for recombinant expression
Spb4_rv_XmaI	ATTTCCCGGGTTATAAGTCGTCAAAATTGCCTTGG	Cloning for recombinant expression
Mak5_fw_BamHI	ATATTAGGATCCGCCATGGGTAAGAAAAGGGCTCCTCAAAAAGG	Cloning for recombinant expression
Mak5_rv_XmaI	AGCGCCCGGGTTAATTATTTCTCTTTTTCTTTTTCTTC	Cloning for recombinant expression
Spb4+500_fw_PstI	ATATACTGCAGCCTGAAGTCTCTCTCCAACAAAATATGATGCGGC	Cloning for yeast complementation
Spb4+500_rv_SmaI	TATATCCCGGGCTACTTCTTAACCTCTAATTACCAACGGGTC	Cloning for yeast complementation
Mak5+500_fw_PstI	TATATATCTGCAGACCTTTAATACTTAACCTGATAATTTTG	Cloning for yeast complementation
Mak5+500_rv_NotI	TATATATGCGGCCGCAAGTCGTTTGCTATTGACAAAG	Cloning for yeast complementation
Spb4_DEAD_fw	GCATGCAGTATGGTAGTTATGGATCAGGCAGACAGATTGTTGGATATGAG	Site-directed mutagenesis
Spb4_DEAD_rv	CTCATATCCAACAATCTGTCTGCCTGATCCATAACTACCATACTGCATGC	Site-directed mutagenesis
Mak5_DEAD_fw	CGAAAGTAAATACGCTAATCCTTGATCAGGCTGATAGGCTGTTACAAG	Site-directed mutagenesis
Mak5_DEAD_rv	CTTGTAACAGCCTATCAGCCTGATCAAGGATTAGCGTATTTACTTTCG	Site-directed mutagenesis
Spb4_SAT_fw	GTCTATTTGCGGCCGCCATGCGTAGCGCCGGATCAGATATCTTTAAGAC	Site-directed mutagenesis
Spb4_SAT_rv	GATCCGGCGCTACGCATGGCGGCCGCAAATAGACCCGTCCTTCTTTGTTTAGGC	Site-directed mutagenesis
Mak5_SAT_fw	GGCAAACTTTGATCTTTGCGGCCGCCTTCTCCATCGACTTGTTTGATAAGC	Site-directed mutagenesis
Mak5_SAT_rv	GCTTATCAAACAAGTCGATGGAGAAGGCGGCCGCAAAGATCAAAGTTTGCC	Site-directed mutagenesis
25S-H39	GAGCGTGTATTCCGGCACC	Structure probing
25S-H63	GCAGTCCACAAGCACGCCCGC	Structure probing
25S-H101	CAAATCAGACAACAAAGGC	Structure probing
ITS1	CGGTTTTAATTGTCCTA	Northern blot probe
ITS2	TGAGAAGGAAATGACGCT	Northern blot probe

pGAL1_SPB4_fw	GCCCATTGATTTGAGGTGTAGTAAGATAATATTAAAAGCTCAGCAGCAATAAAAACATCGAGCTCGTTTAAAC	Yeast strain generation
pGAL1_SPB4_rv	CCTTATCCAGGGAAGTAAAGAAAACCCGAGATTATCCCATTCCAATGACTTTGACATGCACTGAGCAGCGTAATCTG	Yeast strain generation
pGALS_HAS1_fw	GGATAACTGTATACTATAATAATTAGATAAGCTGAGCAATATTAACAGGAGAAGTATGCGTACGCTGCAGGTCGAC	Yeast strain generation
pGALS_HAS1_rv	CTACTACAGGTTCTTCTGTAGATTCAGAATCTCTAGAACGTTTATTTGACGGGGTAGCCATCGATGAATTCTCTGTCG	Yeast strain generation
SPB4-HTP_fw	AAACGGAAGAAAGTTTCCAGCAAAGCTATCCAAGGCAATTTTGACGACTTATCCATGGAGCACCATC	Yeast strain generation
SPB4-HTP_rv	TCATCCATTGGTTAAGAATGTTGAGTGATTCTACGAACAAGGTAACTTTTTTTCCATAATACGACTCACTATAGGG	Yeast strain generation
MAK5-HTP_fw	TGAAAAGACAAACGCCTTAGAAACTTTGAAGAAAAAGAAAAAGAGAAATAATTCCATGGAGCACCATC	Yeast strain generation
MAK5-HTP_rv	ACCGTGTATATTACGTAGAAAATACAGTGAAAGGAGAGTTTCTCTTCAAAAGCCTCGAGATACGACTCACTATAGGG	Yeast strain generation
HAS1-HTP_fw	GGTGCCAGTGGAAAAACGCCAAATACCAAAAGACGTAAAACTCATAAGTCCATGGAGCACCATC	Yeast strain generation
HAS1-HTP_rv	GTGTTGGTTCCACGTACACATTAATAGCATTAAAAATTGATCCTCTGAAATACGACTCACTATAGGG	Yeast strain generation
ERB1-HTP_fw	GCTTGGCTGTTTTCTGCCGGTGCTGATAATACGGCTCGCTTATGGACCACCTCCATGGAGCACCATC	Yeast strain generation
ERB1-HTP_rv	CGAGTGTTTGATTTTTTCTTCTTGGGCGTTATTATACAATATCTACTTCAAATTATTTATTACGACTCACTATAGGGCGA	Yeast strain generation
RLP24-HTP_fw	GCTAAAGAACAGAAGAAGAAATACAAAGAAAATTGCTTTTTCCATGGAGCACCATC	Yeast strain generation
RLP24-HTP_rv	GAGAAAAGAAAGGAAAGAGGCAATTAGCCCGGTTCTGTTCTACGACTCACTATAGGGCG	Yeast strain generation
TIF6-TAP_fw	CCAGAATCCATTTCAGGTAACCTACGTGATACTTTGATTGAAACCTACTCACGTACGCTGCAGGTCGAC	Yeast strain generation
TIF6-TAP_rv	CGCATACAACTGTAAACAGACTTGAGGAAGGAGGGGAATCCCCTCAGGAGTACCATCGATGAATTCGAGCTCG	Yeast strain generation
ARX1-GFP_fw	GCAGAAGAGTGTTGAGACATCAAATGGCGGAGTTGAAGAAACCATGAAAATGCGTACGCTGCAGGTCGAC	Yeast strain generation
ARX1-GFP_rv	CATTTTATGATATACTTATATTATTTATATACTAGCTTTAGAAATGATGAAGTTTCATCGATGAATTCGAGCTCG	Yeast strain generation
U3a/b	GGTACAAATGGCAGTCTGAC	snoRNA qPCR
U14	TTCTTTAGAGACCTTCCTAGG	snoRNA qPCR
U18	TGACAAAAGAGATGTGGTTGAC	snoRNA qPCR
U24	GAGACATACCAATTATCACCAAG	snoRNA qPCR
snR3	GTTTTGATTAGCTGAATGAGAC	snoRNA qPCR
snR4	CCTTTATAGCGGTGCTTTAAC	snoRNA qPCR
snR5	ATTGGTTCGCTCTAGGTGTAC	snoRNA qPCR
snR8	ATCGGTACTGCGCGAGTGAG	snoRNA qPCR
snR9	ACCTATCATTAGTCCTTCAGAC	snoRNA qPCR
snR10	TCTGTCGTCTGTTTTTTAGCAG	snoRNA qPCR
snR11	AAGAAAGTGAGTGGATCTTCCC	snoRNA qPCR
snR13	GTGTGGAAAAACTCAAGCTAC	snoRNA qPCR
snR30	CATTTGGGTAAAACCATACTG	snoRNA qPCR

snR31	TAAACACCTGATACAGTTGGTC	snoRNA qPCR
snR32	GAAATGAGATATTGGGAATCAG	snoRNA qPCR
snR33	AATTGATATAGAAGTGTGTGGAC	snoRNA qPCR
snR34	TGTCTCAAACGAGGCGATAG	snoRNA qPCR
snR35	CAAGGGCTGGTAGGACAGAC	snoRNA qPCR
snR36	GCTATTTTTATCTCACGGTATC	snoRNA qPCR
snR37	CCTAAGCGACTCTTCTTCATG	snoRNA qPCR
snR38	CTGAATGGGTAATAATAGGTAACC	snoRNA qPCR
snR39	GCTGTCGTAACTTATCACCA	snoRNA qPCR
snR39b	ATGTTGTCAACTTAAATTACACC	snoRNA qPCR
snR40	AAGTTTCAGCCTTGTATGAG	snoRNA qPCR
snR41	CCTTTTTCGTTAAGTTTCAG	snoRNA qPCR
snR42	GTTAAGCGACCCATGAAATG	snoRNA qPCR
snR43	AATCTCTGGGTTGTTTAGATG	snoRNA qPCR
snR44	TCCATTACCGTTTACTTTTCC	snoRNA qPCR
snR45	GCAACCCATTGATCTTGTTAC	snoRNA qPCR
snR46	ATAAAGTTGTGCTATTTCCATG	snoRNA qPCR
snR47	ACAATAGCTTTTTAACACTG	snoRNA qPCR
snR48	ATGTTAGGATGTGAAGTTTAAGTAC	snoRNA qPCR
snR49	AAGATTTATCTCTTTTGTCCATC	snoRNA qPCR
snR50	CTTTACAGAACCGCTACACTG	snoRNA qPCR
snR51	TAGATTGGTCTCTTTAACGAAGG	snoRNA qPCR
snR52	GACATTAGCGTGAACAATCTC	snoRNA qPCR
snR53	TGATTAAAATTGTTGTTTACGC	snoRNA qPCR
snR54	CGATCTTGTAGAGAACTTTTACTC	snoRNA qPCR
snR55	CACAATCGTCTTTTTTTTATCC	snoRNA qPCR
snR56	ACACAGACCTGTACTGAACTTTTC	snoRNA qPCR
snR57	ATTTTTCTGAGGAAGTATATGC	snoRNA qPCR
snR58	CTCCTATGGAAGAGAAACTC	snoRNA qPCR
snR59	AATCACCATCTTTCGGCTGAC	snoRNA qPCR
snR60	CAACTGATTGAACATACTATCG	snoRNA qPCR
snR61	AAGATAACCAATTTTACCAAAG	snoRNA qPCR
snR62	ATGGAAGATATACGACTATCAAC	snoRNA qPCR
snR63	GAGTCTTTTAATGTGATGAGTGG	snoRNA qPCR
snR64	TAAAGCCCAGTTTTTAGTAGAG	snoRNA qPCR
snR65	AACTTTATGATTACAGTGTTTTC	snoRNA qPCR
snR66	GAGATTGCTTTTTTATTACTGAC	snoRNA qPCR
snR67	GATTTTACAAACAACAAACACTG	snoRNA qPCR
snR68	CGAGGAAATTGACTCTTAACAG	snoRNA qPCR
snR69	AAAGGGAGAAGATTTTTTTGTC	snoRNA qPCR
snR70	TGATTGGTCACAAGACATCTG	snoRNA qPCR
snR71	ATTTCCTATCCAACATTCATC	snoRNA qPCR
snR72	GAGAACATCAATGAAGAAAACG	snoRNA qPCR
snR73	TGTGACAGGGCGTGGTACTG	snoRNA qPCR
snR74	GAAACAAATTACTCAAATAGACAAG	snoRNA qPCR
snR75	AACTATTAAAATTACCATTCATGC	snoRNA qPCR
snR76	TCCTTTCAAATGAGTGACAATG	snoRNA qPCR
snR77	GAGTATATGTTGATACGTTTTTGC	snoRNA qPCR
snR78	AGTTTCTGAATCTTTTGTGATTAG	snoRNA qPCR
snR79	CAAGACTACAACGGTATCTG	snoRNA qPCR
snR80	GGTTATATTAGTCCATTTCATAGC	snoRNA qPCR
snR81	ACGCTTTTTCACATCTTCTTG	snoRNA qPCR
snR82	TATAGTTTGATAGTAGATGGGCG	snoRNA qPCR
snR83	CCCGATTTGTATTTTATTTTTC	snoRNA qPCR
snR84	AGGAACATGACTCAAAGAGACAC	snoRNA qPCR
snR85	TACCATATAGAGGTGTCAAGTACAC	snoRNA qPCR
snR86	AGAATGTAGTTTCATACCCG	snoRNA qPCR
snR87	TGTTCTATATGGGTGATTAGCG	snoRNA qPCR
snR161	TCAGGCTGTATTTCATAACACTAC	snoRNA qPCR
snR189	ACTTTCAAGTACTTCACACG	snoRNA qPCR

snR190	GAAAAGATGTTGCTTCTGTGAC	snoRNA qPCR
snR191	TATGTTCGTAGTAAAGATCCTCAC	snoRNA qPCR
snoRNA_rv	CGAATTCTAGAGCTCGAGGCAGG	snoRNA qPCR

Supplementary Table 6. Primary antibodies used in this study.

Target	Source
HA	Biolegend (901503)
PAP	Sigma-Aldrich (P1291)
Pgk1	ThermoFisher (22C5D8)
GFP	Roche (11814460001)
Rpl15	Aviva Systems Biology (ARP65141_P50)

SUPPLEMENTARY METHODS

Fluorescence anisotropy measurements

Fluorescence anisotropy experiments[1] were performed using recombinant His_{10}-ZZ-tagged $Mak5_{WT}$, $Mak5_{DEAD}$, $Mak5_{SAT}$, $Spb4_{WT}$, $Spb4_{DEAD}$ and $Spb4_{SAT}$ that had been dialysed against a buffer containing 30 mM Tris-HCl pH 7.5 and 50 mM NaCl. Recombinant proteins at different concentrations (0-4 μM) were mixed with an Atto647-labelled 11 nucleotide RNA (5'-GUAAUGAAAGU-3') at a concentration of 20 nM and incubated at room temperature for 1 min. Anisotropy measurements were performed in a FluoroMax-4 spectrofluorometer (Horriba Scientific) at 30 °C. The obtained data were analysed using the following equation:

$$r = r_0 + \frac{\Delta r_{max}}{[RNA]_{tot}} \cdot \left(\frac{[protein]_{tot} + [RNA]_{tot} + K_d}{2} - \sqrt{\left(\frac{[protein]_{tot} + [RNA]_{tot} + K_d}{2} \right)^2 - [protein]_{tot}[RNA]_{tot}} \right)$$

where r_0 is the anisotropy of free RNA, $\Delta rmax$ is the amplitude, and $[protein]_{tot}$ and $[RNA]_{tot}$ are the total protein and total RNA concentrations respectively. Dissociation constants were calculated using Origin 8.2 software.

Analysis of pre-ribosomal complexes by mass spectrometry

Yeast strains expressing HTP tagged (Spb4) or TAP tagged (Has1 or Mak5) RNA helicases were grown in exponential phase before harvesting. Cell pellets were resuspended in Lysis buffer (50 mM Tris-HCL pH 7.8, 150 mM NaCl, 1.5 mM $MgCl_2$, 0.05% NP-40, 2 mM DTT) and

protease inhibitors. Cells were then lysed by grinding in liquid nitrogen and cell debris were pelleted by centrifugation. Complexes were first immobilised on IgG sepharose and after thorough washing steps, were eluted using TEV protease. Eluates derived from cells expressing TAP tagged proteins were supplemented with 2 mM $CaCl_2$ and 1 mM imidazole, and then incubated with calmodulin beads. Thorough washing steps were performed and complexes were eluted with Lysis buffer supplemented with 1 mM imidazole and 5 mM EGTA. Alternatively, IgG eluates derived from cells expressing HTP tagged proteins were directly incubated with NiNTA and after washing steps, complexes were eluted using a buffer containing 50 mM Tris-HCl pH 7.8, 50 mM NaCl, 150 mM imidazole, 0.1% NP-40 and 5 mM β-mercaptoethanol. Proteins in the final eluates were precipitated by addition of TCA to a final concentration of 20%, separated by denaturing polyacrylamide gel electrophoresis and analysed by mass spectrometry as previously described[2]. In brief, relevant lanes were excised, fragmented and proteins were digested with trypsin before nanoLC-MS/MS analysis. Peak lists were extracted from the raw data using Raw2MSMS software and proteins were identified using MASCOT 2.4 software (Matrixscience) and compared to the UniProtKB *S. cerevisiae* proteome version 2016.01. The fold enrichment of proteins with each of the helicases was determined by calculating a ratio between the number of spectral counts (unique peptides) identified in each of the samples compared to the relevant control samples (wild-type yeast).

Isolation of pre-ribosomal complexes on calmodulin beads

Yeast cells expressing TAP tagged Kre35 were grown in exponential phase, harvested and lysed by grinding in liquid nitrogen in a buffer containing 50 mM Tris-HCl pH 7.8, 150 mM NaCl, 1.5 mM $MgCl_2$, 0.05% NP-40, 2 mM DTT and protease inhibitors. After centrifugation to pellet cell debris and 2 mM $CaCl_2$ and 1 mM imidazole were added. Complexes were retrieved on calmodulin beads that had been pre-blocked using lysis buffer supplemented with 2 mM $CaCl_2$, 1 mM imidazole, 20 mg/mL glycogen, 20 mg/mL BSA and 20 mg/mL *E. coli* tRNA. Following washing steps, complexes were eluted in lysis buffer supplemented with 1 mM

imidazole and 5 mM EGTA. Proteins in the eluate were precipitated by addition of TCA to a final concentration of 20% and then analysed by western blotting using antibodies listed in Supplementary Table 6.

Analysis of snoRNA levels on pre-ribosomes

To determine the relative levels of snoRNAs on pre-ribosomes whole cell extracts from cells expressing or depleted of selected RNA helicases were separated on 10-45% sucrose density gradients by centrifugation in an SW40Ti rotor at 23,000 rpm for 16 h. Fractions containing non-ribosomal and pre-ribosomal complexes were pooled and RNA was extracted. RNAs were polyadenylated using E. coli poly(A) polymerase, a oligo(dT) adaptor was ligated and reverse transcription was performed using Superscript III. RNAs were digested using RNase A and RNase H and quantitative PCR was carried out using a common reverse primer and snoRNA-specific primers (Supplementary Table 5).

SUPPLEMENTARY REFERENCES

1. Kretschmer, J., Rao, H., Hackert, P., Sloan, K.E., Höbartner, C. & Bohnsack, M.T. The m^6A reader protein YTHDC2 interacts with the small ribosomal subunit and the 5'-3' exoribonuclease XRN1. *RNA,* 24, 1339-1350 (2018).
2. Atanassov, I. & Urlaub, H. Increased proteome coverage by combining PAGE and peptide isoelectric focusing: comparative study of gel-based separation approaches. *Proteomics* 13, 2947-2955 (2013).
